# Transglutaminase 2 limits the extravasation and the resultant myocardial fibrosis associated with factor XIII-A deficiency

**DOI:** 10.1016/j.atherosclerosis.2019.12.013

**Published:** 2020-02

**Authors:** Kathryn J. Griffin, Laura M. Newell, Kingsley R. Simpson, Cora M.L. Beckers, Mark J. Drinkhill, Kristina F. Standeven, Lih T. Cheah, Siiri E. Iismaa, Peter J. Grant, Christopher L. Jackson, Richard J. Pease

**Affiliations:** a Discovery and Translational Science Division, Leeds Institute of Cardiovascular and Metabolic Medicine, University of Leeds, Leeds, LS2 9JT, UK; b Bristol Heart Institute, University of Bristol, Bristol, BS2 8HW, UK; c Victor Chang Cardiac Research Institute, Darlinghurst, NSW, 2010, Australia

**Keywords:** Atherosclerosis, Fibrosis, Factor XIII-A, Transglutaminase 2, Myocardium

## Abstract

**Background and aims:**

Transglutaminase (TG) 2 and Factor (F) XIII-A have both been implicated in cardiovascular protection and repair. This study was designed to differentiate between two competing hypotheses: that TG2 and FXIII-A mediate these functions in mice by fulfilling separate roles, or that they act redundantly in this respect.

**Methods:**

Atherosclerosis was assessed in brachiocephalic artery plaques of fat-fed mixed strain apolipoprotein *(Apo)e* deficient mice that lacked either or both transglutaminases. Cardiac fibrosis was assessed both in the mixed strain mice and also in C57BL/6J *Apoe* expressing mice lacking either or both transglutaminases.

**Results:**

No difference was found in the density of buried fibrous caps within brachiocephalic plaques from mice expressing or lacking these transglutaminases. Cardiac fibrosis developed in both *Apoe/F13a1* double knockout and *F13a1* single knockout mice, but not in *Tgm2* knockout mice. However, concomitant *Tgm2* knockout markedly increased fibrosis, as apparent in both *Apoe/Tgm2/F13a1* knockout and *Tgm2/F13a1* knockout mice. Amongst *F13a1* knockout and *Tgm2/F13a1* knockout mice, the extent of fibrosis correlated with hemosiderin deposition, suggesting that TG2 limits the extravasation of blood in the myocardium, which in turn reduces the pro-fibrotic stimulus. The resulting fibrosis was interstitial in nature and caused only minor changes in cardiac function.

**Conclusions:**

These studies confirm that FXIII-A and TG2 fulfil different roles in the mouse myocardium. FXIII-A protects against vascular leakage while TG2 contributes to the stability or repair of the vasculature. The protective function of TG2 must be considered when designing clinical anti-fibrotic therapies based upon FXIII-A or TG2 inhibition.

## Highlights

• Double transglutaminase 2 and Factor XIII-A knockout exacerbates cardiac fibrosis.
• Double knockout does not promote the growth of, or destabilise, brachiocephalic plaques.
• FXIII-A in resident cardiac macrophages does not protect against cardiac fibrosis.
• FXIII-A in inflammatory macrophages may contribute to protection against fibrosis.
• Transglutaminase 2 and Factor XIII-A protect against extravasation of blood.

## Graphical abstract

**Figure:**
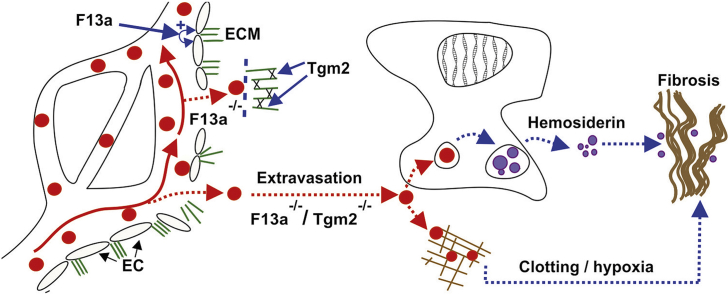

## 1 Introduction

Transglutaminases (TGs) comprise a family of 8 enzymes that catalyse the Ca^2+^-dependent formation of N^ε^ (γ-glutamyl) lysine isopeptide cross-links between and within protein chains [1].

TG2, which is also known as tissue transglutaminase, is expressed in all cell-types and in all tissues, but its expression increases under conditions of stress [2]. Within the cell, TG2 is primarily cytosolic, but separate pools of TG2 exist in the nucleus, endosomes and associated with mitochondria [2]. Although its catalytic activity is strongly suppressed by low Ca^2+^ concentrations and a high [GTP]/[GDP ratio] [1], TG2 has nevertheless been implicated in intracellular signalling [3]. TG2 can also be secreted from the cytosol onto the cell-surface and into the extracellular matrix [4]. Here, in the reduced form [5], TG2 cross-links matrix proteins including collagen I chains [4]. Once oxidised, TG2 functions instead as a structural protein [6,7]. Despite various roles previously attributed to TG2, the *Tgm2* knockout mouse obtained by Iismaa and Graham develops normally but shows an impaired response to stress or injury [8,9]. For example, *Tgm2* knockout compromises the inward arterial remodelling that occurs in response to flow restriction [10].

The consequences of TG2 deficiency in humans are unknown. However, it has been suggested that TG2-mediated cross-linking occurs predominantly in damaged or ageing human tissues [11]. Amongst affected tissues, TG2 is strongly expressed in human atherosclerotic plaques, where it may contribute to plaque stability. However, neither we [12] nor Van Herck et al. [13] observed any difference in the rate of plaque rupture when comparing *Apoe*/*Tgm2* knockout mice with *Apoe* knockout mice.

Blood clotting factor FXIII-A is present as a dimer within the cytosol of megakaryocytes, platelets and resident macrophages of the heart, arteries, lung and placenta [14]. Resident macrophages mediate the release of FXIII-A into the plasma [15], where it circulates as a heterotetrameric zymogen complex with the non-catalytic B-subunit. Following activation by thrombin, FXIII-A cross-links proteins including fibrin and α-2 antiplasmin within blood clots [16] and fibronectin within the extracellular matrix of injured tissues. Deficiency of FXIII-A in human subjects predisposes to cerebral haemorrhage and haemorrhage during pregnancy.

Fatal haemorrhage occurs spontaneously in male *F13a1* knockout mice [17]. and also in pregnant female *F13a1* knockout mice [18]. Non-fatal myocardial bleeds also occur in *F13a1* knockout mice and result in the extravasation of red blood cells into the myocardium [17]. Thus, together with tissue factor and clotting factor VII [19,20], FXIII-A may establish a haemostatic barrier that protects cardiac blood vessels against extravasation under the mechanical stress of beating and reduces the consequent development of fibrosis [19]. Other studies have reported that FXIII-A reduces the permeability of the vasculature [21], but it remains unclear how this observation relates to its protective role against extravasation and fibrosis in myocardial vessels.

Fibrosis is classified as two major types. Replacement fibrosis occurs when collagen deposition forms a scar to fill and stabilise a region within which cells have died, for example the ischaemic area of an infarcted heart [22]. In contrast, interstitial fibrosis results from increased deposition of collagen between living cells, for example amongst cells peripheral to an infarct following a myocardial infarction [22]. Fibrosis is poorly reversible, and in the heart it predisposes to ventricular dysfunction and poorer clinical outcomes [23,24]. Therefore, it would be desirable to limit the extent of fibrosis to reparative scar formation only and minimise the involvement of adjacent healthy tissue. Various stimuli may induce fibrosis following infarction, including the presence of blood within the myocardium [23,24]. TG2 promotes the response to fibrotic stimuli by several proposed mechanisms that involve the profibrotic mediator TGF-β.^25^These mechanisms may include activating the TGF-β precursor complex in the extracellular matrix [25], and directly potentiating the effect of TGF-β signalling on cardiofibroblasts [26]. Consequently, in model systems including the heart [26], TG2 inhibition reduces fibrosis.

Previous studies have suggested that FXIII-A and TG2 act redundantly to mediate functions including arterial remodelling [10] and bone deposition [27]. To investigate potential redundancy, we [21] and Mousa et al. [28] have previously generated mice that are knockouts for both transglutaminases. In this work, we have used mice lacking either or both transglutaminases to determine whether FXIII-A and TG2 act redundantly to stabilise atherosclerotic plaques. We have also used these mice to investigate whether concomitant *Tgm2* knockout reduces fibrosis in the *F13a1* knockout mouse, as might be predicted if TG2 promotes fibrosis in response to extravasated blood.

## 2 Materials and methods

### 2.1 Maintenance of mouse lines

Animal husbandry and procedures were conducted in accordance with guidelines and regulations of the United Kingdom Home Office and of the Universities of Bristol (studies on mixed strain mice) and Leeds (studies on C57BL/6J mice). Mice had free access to food and water, except for limited periods when food was withheld for glucose or insulin tolerance testing.

### 2.2 Generation of mixed strain *Apoe* knockout mice deficient in *F13a1* and/or *Tgm2*

Mixed strain *F13a1*^−/−^ mice [29] were obtained from Harlan Laboratories, Rossdorf, Germany, from a stock bred in recent generations from homozygous knockout females [30]. *Apoe*^−^−/−/*Tgm2*^−/−^ mice on a mixed (C57BL/6J x 129) background [12] were crossed with male mixed strain *F13a1*^−/−^ mice to generate F1 triple heterozygous mice. Interbreeding the F1 mice generated F2 mice including the following genotypes: *Apoe*^−^−/−/*Tgm2*^+/+^/*F13a1*^+/−^ and *Apoe*^−^−/−/*Tgm2*^+/−^/*F13a1*^+/−^. Interbreeding the F2 *Apoe*^−^−/−/*Tgm2*^+/+^/*F13a1*^+/−^ mice generated littermates to examine the effect of FXIII-A deficiency on a TG2 expressing background, while interbreeding the *Apoe*^−^−/−/*Tgm2*^+/−^/*F13a1*^+/−^ resulted in FXIII-A deficiency on a TG2-null background.

### 2.3 Generation of *Apoe* expressing C57BL/6J mice deficient in *F13a1* and/or *Tgm2*

*F13a1* knockout mice on a C57BL/6J background [15] were bred from *F13a1*^+/−^ dams mated either with *F13a1*^+/−^ or *F13a1*^−/−^ sires, since, in agreement with previous findings [18], each of four pregnant C57BL/6J *F13a1* knockout mice suffered prenatal or perinatal mortality. C57BL/6J *F13a1*^−/−^ pups from these crosses were born below Mendelian expectation (226 observed, 325 expected, *p <* 0.001).

C57BL/6J *Tgm2*^+/−^ mice [31] were obtained from a colony maintained as heterozygotes at the Victor Chang Cardiovascular Research Institute Darlinghurst, Australia. C57BL/6J *Tgm2*^+/−^ mice were crossed with C57BL/6J *F13a1*^+/−^ mice to generate *Tgm2*^+/−^/*F13a1*^+/−^ double heterozygous mice and, by further breeding, littermate *Tgm2*^−/−^/*F13a1*^−/−^ (double knockout), *Tgm2*^+/+^/*F13a1*^−/−^ (*F13a1* knockout), *Tgm2*^−/−^/*F13a1*^+/+^ (*Tgm2* knockout) and *Tgm2*^+/+^/*F13a1*^+/+^ (wild-type, WT) mice. C57BL/6J *Tgm2*^−/−^/*F13a1*^−/−^ pups were also born below Mendelian expectation (246 observed, 331 expected *p <* 0.01), while *Tgm2*^−/−^ mice were born in accordance with Mendelian expectation (80 obtained, 88 expected). Oligonucleotides for genotyping are shown in Supplementary Table 1.

C57BL/6J *F13a1*^flox/flox^ mice (floxed in coding exon 7 of the *F13a1* gene) and progeny from crosses with C57BL/6J *Pf4*-cre and *Cd11b*-cre recombinase-expressing mice, were as described previously [15].

### 2.4 Fat feeding

In an initial study (1), 30 male mixed strain *Apoe* knockout mice (age 6–8 weeks) of each transglutaminase genotype, were maintained on a high-fat rodent diet containing 21% lard and 0.15% cholesterol (SDS, Witham, Essex, UK) for 12 weeks. In a follow-up study (2), separate male and female groups of C57BL/6J *Apoe* expressing mice (n = 6 or 7) per transglutaminase genotype) were maintained either on the same high-fat rodent diet or on a standard chow diet for 12 weeks.

### 2.5 Assessment of atherosclerosis and fibrosis

Mice were anaesthetised either by intraperitoneal injection of sodium pentobarbital (20 mg, Rhone Merieux, Harlow, UK), or by inhalation of 2.5% isoflurane (Zoetis, UK). Blood samples were taken, then mice were exsanguinated. For study (1), mice were perfusion-fixed at arterial pressure using 10% buffered formalin, and then the hearts and brachiocephalic arteries were excised. For study (2), mice were exsanguinated via the *vena cava,* and the intact heart was excised while beating and immersed in phosphate buffered saline containing 4% paraformaldehyde.

To assess atherosclerosis, paraffin sections (3 μm) were taken 60 μm from the ostium of the brachiocephalic artery, and also from the first appearance of all three valve leaflets at the aortic sinus. Serial sections were stained with elastin Van Gieson (EVG) and haematoxylin and eosin (H&E) for analysis of plaque morphometry, picrosirius red (S) for the analysis of collagen and with toluidine blue to detect acidic proteoglycans. To assess myocardial fibrosis, heart ventricle sections from 4 separate transverse planes were stained for collagen deposition using Masson's trichrome stain and picrosirius red, and with Perls' Prussian blue stain to assess hemosiderin deposition. In study (**2**), where the hearts were not punctured prior to excision, sections from 5 separate transverse planes across the ventricles were analysed for the presence of fibrosis and haemosiderin and results from each plane were combined to determine the fractional affected areas per mouse.

In some cases, rehydrated paraffin sections were subjected to antigen retrieval by boiling in 10 mM citrate buffer pH 6.0. For activated macrophage detection, sections were quenched in 1% H_2_O_2_ in methanol for 30 min, blocked with rabbit serum, then incubated at 4 °C overnight in biotinylated *Griffonia Simplicifolia* lectin I isolectin B4 (GSA 1B4, 10 μg/mL Vector Laboratories Inc.) prior to detection using the Vectastain ABC kit (Vector Laboratories Inc). For TGF-β detection, sections were washed in 0.05% PBS-Tween, quenched in 1% H_2_O_2_, incubated in 4% goat serum for 30 min, washed and incubated with a 1:100 dilution of TGF-β1 antibody (NBP2-22114) plus a ‘mouse-on-mouse’ blocking reagent (ab64259) for 60 min. Slides were washed and the mouse specific HRP/DAB Detection Kit (ab64259) was applied according to the manufacturer's instruction.

### 2.6 Image analysis

Tissue sections from the mice were imaged using a QICAM Fast 1394 camera (Qimaging, Surrey, BC, Canada) and analysed using Image-Pro Plus software (Media Cybernetics, Carlsbad, CA, USA) or using an Olympus BX61WI inverted microscope with an XC10-IR camera under the control of CellSens software (Olympus). Image analysis was carried out in a blinded manner.

To determine the distribution of collagen fibres, tissue sections that had been stained with picrosirius red, were examined using a Zeiss 710 multiphoton microscope (Zeiss). Excitation was achieved at wavelength of 800–860 nm using a Chameleon laser, and the second harmonic signal characteristic of collagen fibres, was collected at 400 nm onto a Zeiss LSM NDD R2 detector (Zeiss), through a 10x objective lens and an EF SP 485 IR++ filter. Collagen images were merged with brightfield images of cell nuclei.

### 2.7 Real time-PCR (RT-PCR)

Hearts or aortas were disrupted in TRIzol (ThermoFisher Scientific) using a TissueLyser II (Qiagen), RNA was precipitated from the aqueous layer using 100% ethanol and DNA removed using the “DNA-free” kit (ThermoFisher Scientific), prior to cDNA synthesis using the High Capacity Reverse Transcription Kit (ThermoFisher Scientific). RT-PCR amplification was performed using the primer pairs shown in Supplementary Table 2 and cycling conditions of 95 °C for 10 min, 45 cycles of 95 °C for 10 s, 60 °C for 1 min. A melting curve was undertaken to ensure that each reaction had generated a single product and in representative cases, the products of RT-PCR reactions were resolved by agarose gel electrophoresis to verify that they were of the expected size. Transcript levels were normalised to the housekeeper mRNA (Rpl32) using the 2^−ΔCt^ method [32].

### 2.8 Statistics

All data is presented as mean ± 2x standard error. All values were analysed using Prism 7 (GraphPad software, San Diego, CA, USA). Comparisons between each group were carried out using unpaired student t-tests (with Welch's correction) or one-way ANOVA (with Bonferroni correction) for multiple groups. A Kruskal-Wallis test with Dunn's multiple comparison test was used for any data that were not normally distributed; normality was assessed using Shapiro Wilk's testing. Mortality rates and breeding frequencies were analysed using a Chi-squared test. Significance was accepted where *p <* 0.05.

## 3 Results

The results obtained from the various studies below are summarised in Supplementary Table 3.

### 3.1 Fat-feeding is associated with increased mortality in mixed strain, *Apoe* knockout mice that are deficient in FXIII-A

To determine whether FXIII-A and TG2 act redundantly to influence plaque development and stability, transglutaminase knockout mice were crossed with *Apoe* knockout mice and subjected to a 12 week period of fat feeding to induce atherosclerosis.

Unexpectedly, mortality over the feeding period was increased in both *Apoe*/*F13a1* double knockout (5/38, *p* = 0.02) and *Apoe*/*Tgm2*/*F13a1* triple knockout mice (8/39, *p* = 0.007) relative to *Apoe* knockout mice (1/49). Mortality was not increased in *Apoe*/*Tgm2* double knockout mice (1/46). Body weights of mice at 6–8 weeks of age were similar between genotypes. Final body weights were lower in *Apoe*/*Tgm2* double knockout and *Apoe*/*Tgm2*/*F13a1* triple knockout mice (Supplementary Figure I). As expected, plasma levels of total cholesterol increased in fat-fed *Apoe* knockout mice of all genotypes. In addition, there was an unanticipated additional increase in HDL cholesterol in *Apoe*/*Tgm2* double knockout and *Apoe*/*Tgm2*/*F13a1* triple knockout mice which was accompanied by a decrease in plasma triacylglycerols. (Supplementary Figure II).

### 3.2 Atherosclerotic plaque stability is unaffected in mixed strain *Apoe* knockout mice that are also deficient both in FXIII-A and TG2

Atherosclerosis was assessed at two independent sites in mice that had survived fat-feeding: brachiocephalic artery plaques which show features of advanced atherosclerosis and mouse aortic retrovalvular sinus plaque lesions which develop as simple xanthomas.

Brachiocephalic plaques of *Apoe*/*Tgm2*/*F13a1* triple knockout mice showed a 28% decrease in cross-sectional area compared to *Apoe* knockout controls (*p* = 0.047) but plaque area was unaltered in both *Apoe*/*F13a1* and *Apoe*/*Tgm2* knockout mice (Table 1). Acidic proteoglycan deposition (indicating areas of chondrocytic metaplasia which can progress to calcification), was also reduced within plaques of *Apoe*/*Tgm2*/*F13a1* knockout mice (Table 1), suggesting that the smaller plaques had not advanced as far as the plaques in other genotypes. The frequency of buried fibrous caps per unit plaque area (an index of the rate of plaque rupture) [33] did not differ across the groups (Table 1). However, plaque collagen content was reduced by 30% in *Apoe* knockout mice lacking either TG2, or, both TG2 and FXIII-A. Staining with GSA 1B4 showed no difference in the density of activated macrophages between plaques from mice of different genotypes.

**Table 1:** Atherosclerotic plaque morphometry in mixed strain *Apoe* knockout mice deficient in transglutaminases TG2 and or FXIII-A.

	Parameter	*Apoe*^−/-^(n = 30)	*Apoe*^−/-^/*F13a1*^−/−^(n = 28)	*Apoe*^−/-^/*Tgm2*^−/−^(n = 30)	*Apoe*^−/-^/*Tgm2*^−/−^/*F13a1*^−/−^(n = 27)
Brachiocephalic artery	Plaque area (x 10^3^ μm^2^)	106 ± 8	106 ± 7	102 ± 7	76 ± 10*
	Buried fibrous caps (per mm^2^ plaque area)	14.8 ± 2.4	13.8 ± 1.7	12.5 ± 3.2	11.5 ± 2.5
	Elastin content (%)	17.2 ± 1.6	13.1 ± 1.9	11.0 ± 1.2	13.0 ± 2.2
	Lipid content (%)	13.9 ± 1.6	11.7 ± 1.8	16.1 ± 1.6	15.5 ± 2.5
	Medial collagen content (%)	18.5 ± 2.7	22.1 ± 3.0	18.0 ± 2.8	11.4 ± 1.6
	Plaque collagen content (%)	29.2 ± 2.2	31.2 ± 2.0	21.5 ± 2.1***	19.6 ± 2.4***
	Plaque acidic proteoglycan content (%)	10 ± 2	8 ± 1	9 ± 2	3 ± 1**
	Plaque lectin staining^a^	19.0 ± 3.1	16.7 ± 2.4	21.5 ± 4.0	24.2 ± 3.9
Aortic sinus	Vessel area (x 10^3^ μm^2^)	1284 ± 60	1254 ± 63	1351 ± 51	1239 ± 75
	Plaque area (x 10^3^ μm^2^)	59 ± 5	52 ± 4	65 ± 4	62 ± 6
	Elastin content (%)	11 ± 1	10 ± 2	8 ± 1	9 ± 1
	Lipid content (%)	6 ± 1	5 ± 1	8 ± 1	9 ± 1
	Collagen content (%)	54 ± 5	55 ± 2	54 ± 4	58 ± 1
	Acidic proteoglycan content (%)	10 ± 3	10 ± 4	14 ± 4	13 ± 2

Brachiocephalic plaque area (*p < 0.05), content of acidic proteoglycan (**p < 0.01) and collagen (***p < 0.001) differed between genotypes. Other parameters were unchanged.

a Plaques were stained with Griffonia Simplicifolia lectin I isolectin B4 (GSA 1B4) to detect infiltration of activated macrophages.

We also examined atherosclerosis in the aortic sinus. However, although it has been observed that knockout of TG2 within macrophages increases lesion size at this site [34], no statistically significant differences between genotypes were observed in size or composition of aortic sinus plaques (Table 1).

### 3.3 Cardiac fibrosis due to FXIII-A deficiency is exacerbated, rather than reduced, by concomitant TG2 deficiency in *Apoe* knockout mice

TG2 is profibrotic in various models [25]. We therefore measured fibrosis in the hearts of mice that survived the 12 week fat-feeding period to determine whether *Tgm2* knockout decreased the fibrotic response associated with *F13a1* knockout.

**Fig. 1:**
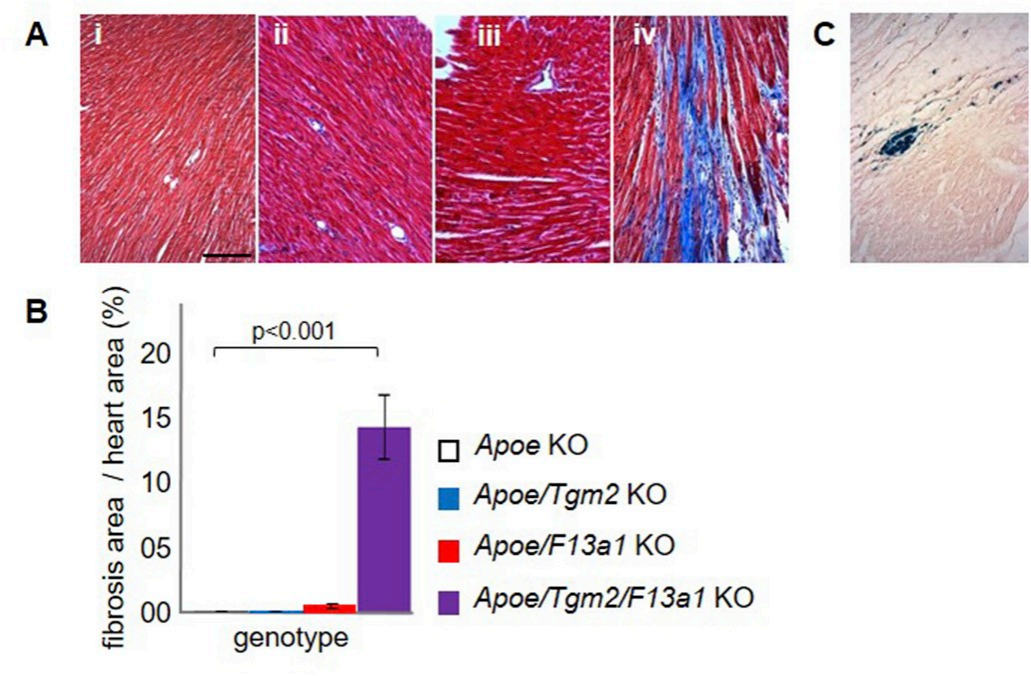
Cardiac fibrosis in *Apoe* knockout mice deficient in transglutaminases TG2 and/or FXIII-A. (A) Representative Masson's (M) trichrome-stained sections of left ventricle wall from (i) *Apoe* knockout (KO), (ii) *Apoe*/*F13a1* knockout, (iii) *Apoe*/*Tgm2* knockout, and (iv) *Apoe*/*Tgm2*/*F13a1* knockout mice. Scale bar represents 100 μm. (B) Quantitation of fibrosis in mice of the various genotypes (n = 30 per genotype, except *Apoe/Tgm2/F13a1* knockout where n = 28) determined by the area of the ventricle stained by Masson's trichrome. (C) Fibrotic heart section from an *Apoe*/*F13a1* knockout mouse which was stained with Perls' Prussian blue to detect haemosiderin deposits. Scale bar as in (A). The section was counterstained with Neutral Red. (For interpretation of the references to colour in this figure legend, the reader is referred to the Web version of this article.)

Contrary to expectation, 3/30 *Apoe*/*F13a1* double knockout mice and 20/28 *Apoe*/*Tgm2*/*F13a1* triple knockout mice showed myocardial fibrosis (*p <* 0.0001, Fig. 1A). Further, the extent of ventricular fibrosis was less in the three affected *Apoe*/*F13a1* double knockout mice (4.0 ± 0.5%) than in the 20 affected *Apoe*/*Tgm2*/*F13a1* triple knockout mice (17.8 ± 9.4%, *p <* 0.01). The percentage fibrotic area per ventricle (Fig. 1B), averaged across both fibrotic and non-fibrotic mice of each genotype, increased approximately 30-fold between the *Apoe*/*Tgm2*/*F13a1* triple knockout mice and the *Apoe*/*F13a1* double knockout mice. The presence of haemosiderin within the fibrotic myocardium (Fig. 1C) suggested that extravasation of red blood cells had occurred.

### 3.4 Cardiac fibrosis due to FXIII-A deficiency is exacerbated by concomitant TG2 deficiency in C57BL/6J *Apoe* expressing mice

Traits associated with gene knockout may be expressed variably in mice of different genetic backgrounds [35,36]. To confirm that *Tgm2* knockout exacerbated the fibrosis associated with *F13a1* knockout, and might therefore provide a reproducible model of fibrosis, we repeated the study using mice on a defined C57BL/6J background. These mice were either recently derived (*F13a1* knockout), or had been maintained as heterozygotes (*Tgm2* knockout), to avoid the selection of modifying traits. These mice also expressed *Apoe* to avoid possible effects of *Apoe* knockout upon, for example, vascular permeability [37] or macrophage survival [38,39].

In contrast to the mixed strain *Apoe*/*Tgm2*/*F13a1* triple knockout or *Apoe*/*F13a1* double knockout mice, spontaneous deaths did not occur amongst either the C57BL/6J *F13a1* single knockout or *Tgm2*/*F13a1* double knockout mice, whether on a high fat or chow diet. No significant differences in body weight gain or plasma metabolite levels were apparent between genotypes at termination (Supplementary Figures I and II.)

**Fig. 2:**
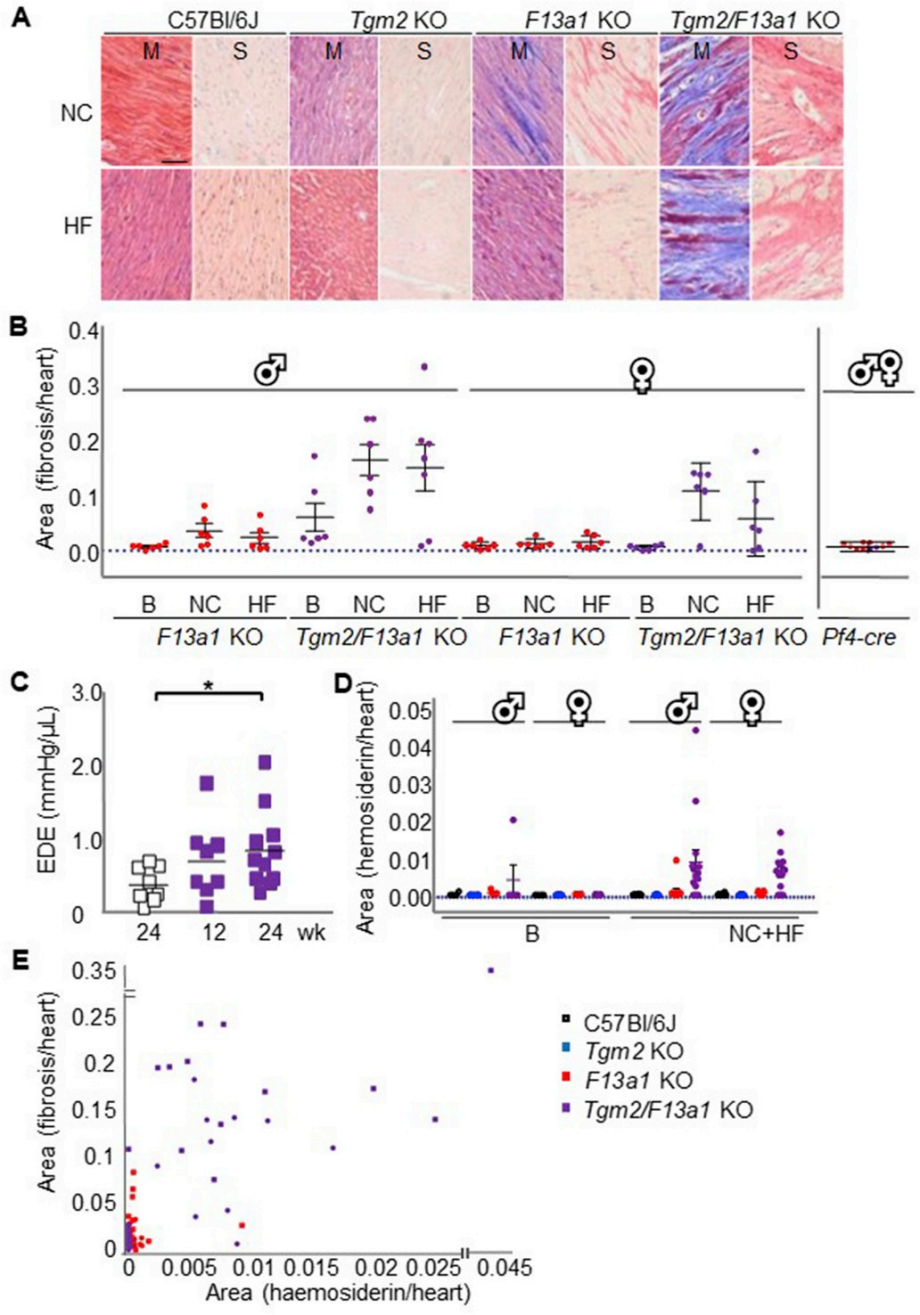
Cardiac fibrosis in *Apoe* expressing C57BL/6J mice deficient in transglutaminases TG2 and/or FXIII-A. (A) Representative sections of left ventricle stained with Masson's trichrome (M) or Picrosirius Red (S) from 20 week old male *Apoe*^+/+^ mice of the genotypes shown that had been maintained on a normal chow diet (NC) or high-fat diet (HF) for 12 weeks, from 8 weeks of age. Scale bar represents 100 μm. (B) Quantitation of fibrosis in 5 ventricular sections from the hearts of 8 week old mice maintained on NC at baseline (B) and then a further 12 weeks of NC or HF diet (n = 6, except male HF *Tgm2/F13a1* knockout mice where n = 7). Fibrosis was exacerbated in *Tgm2/F13a1* double knockout mice as compared to *F13a1* knockout mice, and in male mice relative to female mice, (*p <* 0.05 in each case). Fibrosis was not detected following 20 weeks of normal-chow feeding in *Pf4-cre.F13a1*^*flox/flox*^ mice of either gender (Pf4-cre, n = 10). Fibrosis was not detected in *Tgm2* knockout or C57BL/6J wild-type mice (not shown). (C) End diastolic elastance (EDE) values of hearts from C57BL/6J *wild-type* mice at 24 weeks and from *Tgm2/F13a1* double knockout mice at ages 12 and 24 weeks calculated from the pressure volume parameters shown in Supplementary Figure III. The development of fibrosis in the *Tgm2/F13a1* double knockout mice was verified post-mortem, and was associated with an increase in EDE. (D) Haemosiderin deposition in ventricles of mice of the genotypes indicated at baseline (8 weeks) or after being maintained for a further 12 weeks on either a normal chow (NC) or a high fat (HF) diet. Haemosiderin content was increased in both male and female *Tgm2/F13a1* double knockout mice (*p <* 0.05 in each case). (E) Haemosiderin deposition plotted against the extent of fibrosis in all male (square) and female (circle) F13a1 knockout and Tgm2/F13a1 double knockout mice. The extent of fibrosis was positively correlated with haemosiderin deposition, (r = 0.7041, *p <* 0.0001 n = 73). (For interpretation of the references to colour in this figure legend, the reader is referred to the Web version of this article.)

Cardiac fibrosis was apparent in C57BL/6J *F13a1* knockout and in C57BL/6J *Tgm2*/*F13a1* double knockout mice, but not in *Tgm2* knockout or wild-type mice (Fig. 2A). Cardiac fibrosis at 20 weeks of age was more extensive in C57BL/6J *Tgm2*/*F13a1* double knockout mice than in C57BL/6J *F13a1* single knockout mice, and in both genotypes was more extensive in male than in female mice. However, fibrosis was not increased in mice fed a high-fat diet over chow-fed mice. Cardiac fibrosis was apparent at 8 weeks of age in some *Tgm2*/*F13a1* double knockout mice (Fig. 2B). Cardiac pressure-volume loop measurements were obtained to determine whether fibrosis compromised cardiac function. *Tgm2*/*F13a1* knockout mice fed on standard chow for 24 weeks, showed an increase in left ventricular stiffness relative to wild-type mice (Fig. 2C), consistent with fibrosis, but in general, cardiac function was well preserved (Supplementary Figure III.).

### 3.5 Additional *Tgm2* knockout promotes the development of interstitial fibrosis secondary to increased extravasation of blood

To determine whether concomitant Tgm2 knockout resulted in more extensive fibrosis through increasing the extravasation of blood or by enhancing the fibrotic response to extravasated blood, hemosiderin was quantified in the myocardia of mice of each genotype (Fig. 2D), and the relationship between hemosiderin content and fibrosis was determined. Combining data from the *F13a1* single and *Tgm2*/*F13a1* double knockout mice revealed that hemosiderin deposition positively correlated with fibrosis (Fig. 2E), suggesting that the profibrotic effect of *Tgm2* knockout resulted from increased extravasation.

**Fig. 3:**
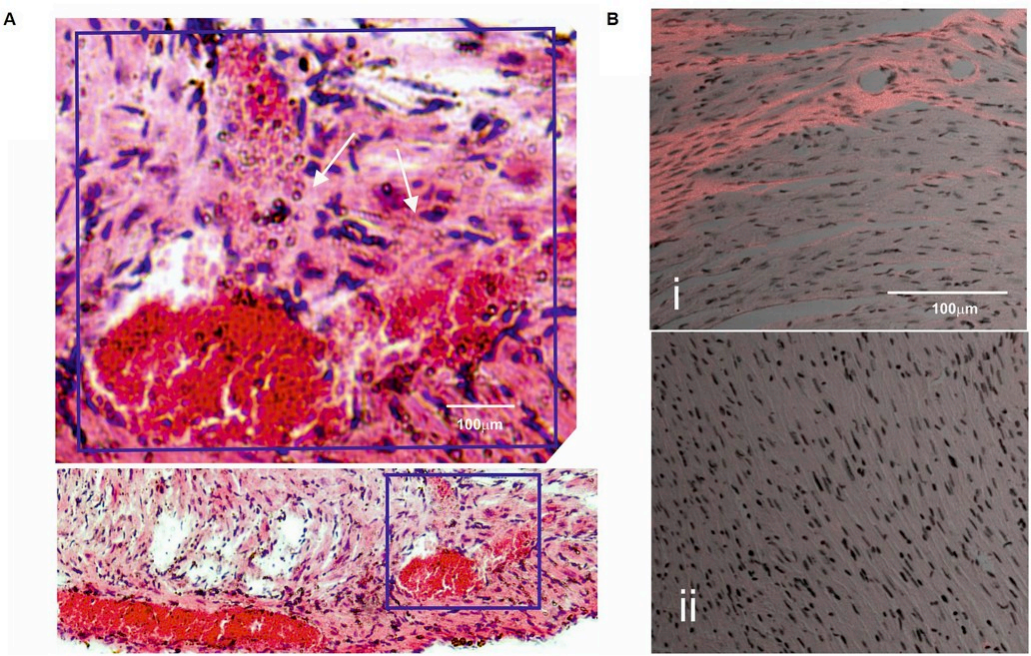
Loss of blood vessel integrity leads to interstitial fibrosis in *Apoe* expressing C57BL/6J *Tgm2*/*F13a1* double knockout mice. (A) H&E stained heart section from a C57BL/6J *Tgm2*/*F13a1* double knockout mouse. Arrowheads show clusters of red blood cells extravasating into the myocardium from an apparently damaged epicardial blood vessel. (B) Representative merged images of collagen (orange) obtained by dual photon microscopy superimposed on brightfield images (shown in greyscale) from (i) a C57BL/6J *Tgm2*/*F13a1* double knockout mouse and (ii) a C57BL/6J wild-type mouse. Elongated nuclei are distributed throughout the fibrotic region indicating a lack of cell death and the presence of predominantly interstitial-type fibrosis. (For interpretation of the references to colour in this figure legend, the reader is referred to the Web version of this article.)

Staining fibrotic myocardia with H&E identified a *Tgm2*/*F13a1* knockout heart where recent vessel damage was evident with escape of red blood cells (RBCs) at this site (Fig. 3A). To determine whether replacement or interstitial fibrosis predominated, sections of *Tgm2*/*F13a1* knockout hearts were stained with picrosirius red and then examined by dual photon microscopy. The second harmonic signal at 400 nm, diagnostic for collagen fibres, was apparent within fibrotic regions of 4 hearts that were examined. Elongated nuclei were distributed throughout the collagen fibres (Fig. 3B). The estimated density of nuclei within two fibrotic areas in the myocardium of a representative *Tgm2*/*F13a1* knockout heart (544 nuclei.mm^−2^) was similar to the density in adjacent uninvolved regions of that same heart (398 nuclei.mm^−2^) and was similar to the density of cells in a representative non-fibrotic wild type heart (418 nuclei.mm^−2^), and a single non-fibrotic *Tgm2*/*F13a1* knockout heart (346 nuclei mm^−2^). Bearing in mind, however, that we observed some differences in the density of nuclei between individual sections within a single healthy heart, we considered that expressing the density of nuclei within fibrotic regions (126 ± 28%, n = 4 hearts, 2 fibrotic sections per heart) relative to the density in adjacent to fibrotic regions (100%) was the more informative comparison. The similar cell density is consistent with the development of interstitial-rather than replacement-type fibrosis.

TGF-β1 frequently mediates profibrotic signalling, and has been implicated in models of cardiac fibrosis [26]. In accord with this, TGF-β antigen was detectable by immunohistochemistry at the margins of fibrotic regions in hearts from *F13a1* knockout and *Tgm2*/*F13a1* double knockout mice, but not in non-fibrotic hearts (Supplementary Figure IV). However, we cannot exclude the possibility that histochemistry may have detected the latent precursor complex in addition to active TGF-β. TGF-β staining in fibrotic hearts was variable, possibly reflecting the episodic development of fibrosis in response to bleeding. In some regions, staining was diffuse, consistent with release of TGF-β into the ECM.

### 3.6 The phenotype at baseline is similar between control and transglutaminase knockout mice

While the results above suggest that TG2 limits the extravasation that results from FXIII-A deficiency, we also investigated whether knockout of transglutaminases might influence the fibrotic response by other mechanisms. If this were the case, it would complicate the use of this model to investigate the response to extravasated blood.

**Fig. 4:**
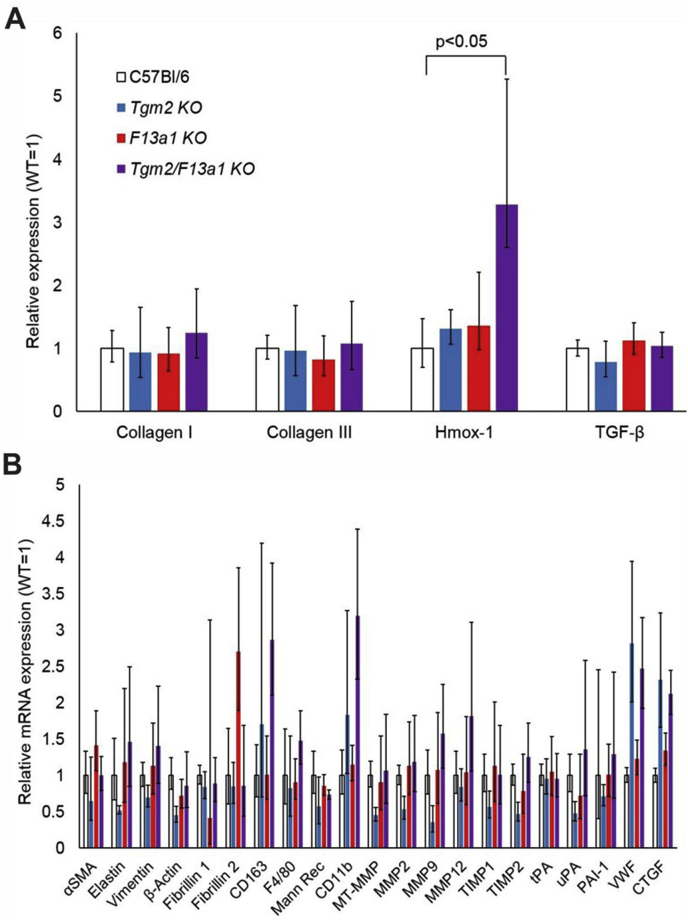
Transcript levels in hearts from *Apoe* expressing mice deficient in transglutaminases TG2 and/or FXIII-A. Levels of transcripts in hearts from 8 week old mice were determined by quantitative PCR. Transcripts encoding the proteins indicated were normalised to the housekeeper transcript, Rpl32 mRNA. Averaged levels of each transcript in transglutaminase knockout mice are shown relative to levels of that transcript in C57BL/6J WT mice. (A) Cardiac mRNAs encoding heme oxygenase 1 (Hmox-1), but not collagens I or III or transforming growth factor β1 (*Tgf-β*), were elevated in the hearts of *Tgm2/F13a1* double knockout mice, (n = 30 control mice, n = 20 transglutaminase knockout mice). (B) Cardiac mRNAs encoding other transcripts were assayed in the hearts of control (n = 20) and transglutaminase knockout mice (n = 10). None of these transcripts showed a significant elevation in the hearts of *Tgm2/F13a1* double knockout mice relative to C57Bl/6J WT mice. (SMA, smooth muscle actin; Mann Rec, C-type mannose receptor; (MT)-MMP, (membrane type)-matrix metalloproteinase; TIMP, Tissue inhibitor of matrix metalloproteinases; (t/u)PA, tissue or urokinase type plasminogen activator; PAI-1, plasminogen activator inhibitor 1; VWF, von Willebrand factor; CTGF, connective tissue growth factor).

It has been reported that TG2 expression negatively regulates the transcription of TGF-β1 and collagens I and III mRNA [40,41], in which case TG2 knockout might increase the expression of each and establish a profibrotic state. However, expression of transcripts encoding collagens I and III and TGF-β1 were comparable between genotypes in mice at 8 weeks of age. Transcripts encoding haemoxygenase were elevated in *Tgm2/F13a1* double knockout mice relative to other genotypes (*p <* 0.05), which may reflect a response to early bleeding in these mice, which presumably preceded the onset of fibrosis (Fig. 4A). We also quantified transcripts encoding other structural proteins, and enzymes mediating extracellular matrix turnover; none of these showed a consistent and significant elevation in *Tgm2/F13a1* double knockout mice relative to other genotypes (Fig. 4B). Further, in agreement with our previous work [30], there was no evidence that induction of TG1 or TGs 3, 4, 5, 6, and 7 compensated for the absence of FXIII-A and/or TG2 in the C57BL/6J mice (Supplementary Figure V.).

Some [42], but not all [31], reports have suggested that glucose metabolism is impaired by TG2 knockout, suggesting that metabolic dysfunction might impact on fibrosis development. We observed that C57BL/6J *Tgm2*/*F13a1* double knockout mice remained viable up to 40 weeks, and that weight gain was not significantly different from other genotypes (Supplementary Figure VIA). Insulin and glucose tolerance were comparable between wild-type and C57BL/6J *Tgm2*/*F13a1* double knockout mice either at 10 weeks or 40 weeks of age (Supplementary Figure VIB.). It has also been suggested that transglutaminases facilitate phagocytosis by macrophages [43], and this could affect the development of fibrosis through several pathways. However we observed no difference using isolated macrophages from wild-type versus C57BL/6J *Tgm2*/*F13a1* double knockout mice (Supplementary Figure VII).

Noll et al. [21] reported that absence of FXIII-A may increase vascular permeability systemically, which could promote RBC extravasation in addition to any effect that focal vessel damage might have. However, while we observed an increase vascular permeability to Evans Blue dye in C57BL/6J *F13a1* knockout mice, we observed no further increase in C57BL/6J *Tgm2*/*F13a1* double knockout mice, suggesting that increased permeability is unlikely to contribute to increased extravasation in these mice (Supplementary Figure VIII). Because the complete survival of C57Bl/6J *F13a1* knockout mice differed from the premature haemorrhagic deaths that occurred in another line of *F13a1* knockout mice [17,29], we confirmed that the clotting deficiency in our C57Bl/6J *F13a1* knockout mice was similar to that reported in the haemorrhagic line [29] (Supplementary Figure IX). This excluded the possibility that rescue of the clotting deficit accounted for the improved survival. We also verified that whole blood clotting parameters were similar between C57Bl/6J *F13a1* knockout and *Tgm2*/*F13a1* double knockout mice, excluding the possibility that *Tgm2* present in blood cells might contribute to clot stability, and that a further decrease in clot stability in *Tgm2*/*F13a1* double knockout mice might account for the increase in fibrosis (Supplementary Figure IX).

### 3.7 FXIII-A within cardiac resident macrophages does not protect against cardiac fibrosis

FXIII-A is present both as a soluble plasma protein and within cells, including cardiac macrophages [15]. To determine whether cardiac macrophage FXIII-A confers protection against fibrosis, we examined the hearts of (*Apoe expressing*) *Pf4*-cre. *F13a1*^flox/flox^ mice maintained on a normal chow diet, to determine whether, as in *Apoe* expressing *F13a1* knockout mice, fibrosis was evident. In previous studies, we established that expression of Pf4-cre abolishes FXIII-A expression within cardiac resident macrophages of *Pf4*-cre.*F13a1*^flox/flox^ mice, whereas conventional myeloid –cre alleles do not [15]. Cardiac fibrosis was undetectable in *Pf4*-cre.*F13a1*^flox/flox^ mice (Fig. 2B, Supplementary Figure X), excluding a protective role for FXIII-A in cardiac macrophages, as well as within platelets which are also depleted of FXIII-A. Plasma FXIII-A activity is reduced to 20%, in *Pf4*-cre.*F13a1*^flox/flox^ mice [15] and it may therefore be that this reduced plasma pool is sufficient to confer protection.

## 4 Discussion

FXIII-A [44] and TG2 [45] are both present within human atherosclerotic plaques, where they may cross-link and stabilise the extracellular matrix. FXIII-A also protects against the development of fibrosis in the mouse heart [17], while TG2 promotes the development of fibrosis in several disease models [25]. We have investigated the protective roles of these two transglutaminases in transglutaminase knockout mice.

Previously, neither we [12] nor Van Herck et al. [13] observed any alteration in the density of buried caps in brachiocephalic plaques in mixed strain *Apoe*/*Tgm2* knockout mice relative to *Apoe* knockout mice, suggesting that the rate of plaque rupture was the same in both genotypes. Here, we have further shown that the density of buried caps is unaltered in male *Apoe*/*Tgm2*/*F13a1* knockout mice relative to *Apoe* knockout mice. Despite this, and in agreement with Van Herck et al. [13] we observed that collagen (which stabilises human plaques [46]) is decreased in brachiocephalic plaques from *Apoe*-deficient *Tgm2* knockout and *Tgm2*/*F13a1* double knockout mice. Thus, while neither FXIII-A nor TG2 is required to generate new caps, or, in the short term, to stabilise existing caps, TG2 may yet prove protective [46,47], possibly through its ability to cross-link collagen I chains [4]. It is possible that expression of another transglutaminase is induced within the brachiocephalic plaque to compensate for the absence of FXIII-A or TG2. While we have not seen evidence for compensation in aortas (Griffin et al.*, in preparation*) or within other tissues [30,31], we cannot exclude compensation in the brachiocephalic artery. However, the disparate activation mechanisms of the various transglutaminases [1], make the possibility of compensation seem unlikely.

Souri et al. [17] observed that male *F13a1* knockout mice deposit haemosiderin and develop cardiac fibrosis, suggesting that FXIII-A contributes to the haemostatic barrier in the cardiac vasculature. The mechanism of protection is uncertain, but is unlikely to be a result of cross-linking fibrin, since fibrinogen knockout mice do not develop fibrosis [19]. We detected mild cardiac fibrosis in a small proportion of fat-fed *Apoe*/*F13a1* double knockout mice, and extensive fibrosis in the majority of *Apoe*/*Tgm2*/*F13a1* triple knockout mice. To confirm that concomitant *Tgm2* knockout exacerbates fibrosis, we bred *F13a1* knockout and *Tgm2*/*F13a1* double knockout mice, each upon a defined C57BL/6J background. We confirmed that fibrosis was exacerbated in *Tgm2*/*F13a1* knockout mice relative to *F13a1* single knockout mice and have further shown that fibrosis was increased in male mice relative to female mice.

Imaging fibrotic regions in the C57BL/6J *Tgm2*/*F13a1* double knockout mice showed that interstitial, rather than replacement, fibrosis predominated. While interstitial fibrosis can impair electrical signalling in the heart [48], cardiomyocytes within the affected region would nevertheless be expected to remain viable and contractile. In agreement with this, pressure volume loop measurements indicated that cardiac function was generally well preserved in the C57BL/6J *Tgm2*/*F13a1* double knockout mice, but with an increase in end diastolic elastance, consistent with ventricular stiffening. The presence of fibrillar collagen in the fibrotic regions suggests that collagens I and III will predominate, although further work will be required to verify this.

We detected haemosiderin in the fibrotic myocardia of both *Apoe*/*Tgm2*/*F13a1* triple knockout and C57BL/6J *Tgm2*/*F13a1* double knockout mice. While this strongly suggests that red blood cells had extravasated and been engulfed by macrophages, a small fresh bleed was detected in the myocardium of only one *Tgm2*/*F13a1* double knockout mouse. Presumably, frequent or significant bleeds would have proved fatal. In contrast, we detected extensive cardiac haemorrhage at termination in a single mouse from the *Apoe*/*Tgm2*/*F13a1* triple knockout line, (Supplementary Figure XIA), amongst which premature deaths occurred. Extravasation of blood cells might occur either because the cardiac vasculature showed increased permeability throughout its length or because discrete breakages had caused leakage from the vasculature. The second possibility is supported by the detection of leakage from a damaged blood vessel in a *Tgm2*/*F13a1* double knockout mouse. Such intermittent bleeding would be expected to cause fibrosis to develop episodically, which is consistent with the finding of macrophage invasion in 5 of 25 *Apoe*/*Tgm2*/*F13a1* hearts examined (Supplementary Figure XIB). Similarly TGF-β staining varied between fibrotic regions, which is again consistent with episodic development. However, it should be noted that immunohistochemistry may not have confirmed the presence of the active, rather than the precursor, form of TGF-β.

We observed that haemosiderin deposition generally correlated with fibrosis in the *F13a1* knockout and *Tgm2*/*F13a1* double knockout mice, (with the constraint that a recent bleed could generate haemosiderin prior to fibrosis, whilst an old bleed could have generated fibrosis that persisted after the haemosiderin was cleared). This suggests that the protective role of TG2 involves strengthening the basal structure of the vessel and/or promoting its repair. We also deduced a role for TG2 in protecting vessels against mechanical stress from parallel carotid ligation experiments. Ligation led to the expected outcome of inward vessel remodelling in *Apoe*/*F13a1* knockout and WT mice. However, ligation led to vessel rupture in *Apoe*/*Tgm2*/*F13a1* triple knockout mice, and to dilatation and elastic lamina breakage without rupture in *Apoe*/*Tgm2* knockout mice (Supplementary Figure XII.).

Administering plasma FXIII-A protects FXIII-A deficient patients against haemorrhage [49]. To determine whether plasma FXIII-A also protects against fibrosis, we recombined the *F13a1*^flox/flox^ gene using *Pf4*-cre which completely ablates FXIII-A expression both within CD163^pos^ resident cardiac macrophages and within platelets. C57BL/6 *Pf4*-cre.*F13a1* ^flox/flox^ mice did not develop fibrosis suggesting that the residual 20% of plasma FXIII-A, is likely to be protective. However we cannot completely exclude a role for macrophage FXIII-A in conferring protection since we detected cardiac fibrosis in a small number of *Cd11b*-cre.*F13a1*^flox/flox^ mice (n = 4), (Supplementary Figure X.), even though these mice have plasma FXIII-A levels 65% of wild-type [15]. It is therefore possible that some protection is conferred by FXIII-A within inflammatory macrophages (the lineage within which *Cd11b* is strongly expressed). If so, this may be similar to the role that FXIII-A in inflammatory macrophages plays in stabilising scars following myocardial infarction [50]. Further work is required to confirm that the *Cd11b*-cre transgene has mediated its effects by suppressing *F13a1* expression, as opposed to exerting off-target effects on other aspects of monocyte/macrophage function.

Male FXIII-A knockout mice are prone to sudden haemorrhagic deaths [17] while female mice may haemorrhage during pregnancy [18]. We observed spontaneous deaths amongst the fat-fed, mixed strain *Apoe*/*F13a1* double knockout and *Apoe*/*Tgm2*/*F13a1* triple knockout mice and, in cases where autopsy was possible, haemorrhage was confirmed. However, the increase in death rates (1.5-fold) in the triple knockout mice was much less than the increase in fibrosis (32-fold). Further, deaths did not occur amongst the C57BL/6J *F13a1* or *Tgm2*/*F13a1* mice post weaning, even up to 40 weeks, despite comparable levels of fibrosis in between *Apoe*/*Tgm2*/*F13a1* triple knockout mice and *Tgm2*/*F13a1* mice. It therefore appears that different factors govern the tendency to small bleeds, which lead to myocardial fibrosis, as opposed to lethal bleeds. Further, even though adult *Apoe*/*Tgm2*/*F13a1* triple knockout mice did not suffer haemorrhage, C57BL/6J *F13a1* knockout pups were born in occasional non-viable litters with evidence of haemorrhage (Supplementary Figure XIII.), again suggesting that additional background factors affect the vascular phenotype associated with FXIII-A deficiency.

In summary, the increase in fibrosis in *F13a1* knockout mice that resulted from concomitant *Tgm2* knockout was novel and unexpected, since TG2 is frequently profibrotic [26,51]. However, it cannot be concluded that TG2 is *anti-fibrotic* in this model. It might simply be that the increased extravasation occurring in double knockout mice has outweighed any moderating effect on fibrotic response associated with the absence of TG2.

Since Tgm2 expression is absent from conception, we cannot be certain whether it has increased extravasation by weakening basal vessel structure or by compromising repair of damaged tissue. If it is the latter, then there may be circumstances in which inhibiting TG2 to reduce the fibrotic response could instead compromise repair and worsen pathology. Certainly, the inhibition of TG2 would seem to be an unsuitable strategy to treat fibrosis in response to red cell extravasation, as occurs after myocardial infarction. It may also be important to ensure that TG2 inhibitors intended for therapeutic use do not show significant inhibition of FXIII-A, if FXIII-A does indeed maintain vessel integrity in damaged or diseased tissues.

Irrespective of this, the reproducible fibrosis that occurs in C57BL/6J *Tgm2*/*F13a1* knockout mice, with excellent survival and satisfactory preservation of cardiac function and other metabolic parameters, offers a robust model to investigate both the toxic effects of red blood cells within the myocardium and novel therapeutic approaches. Further studies will address these possibilities.

## Author contributions

KJG, LMN, KRS, CMLB, MJD, KFS and LTC performed experimental work and ongoing data analysis. SI provided essential resources and valuable advice. PJG, CLJ and RJP prepared funding applications and maintained oversight of the project. KJG and RJP prepared the manuscript for submission along with CMLB.

## Financial support

We thank the British Heart Foundation for Programme Grant support (RG02/29261), for fellowship support to LMN, KJG and KRS (FS/08/050/25667, FS/11/91/29090 and FS/13/36/30243) and for funding the purchase of the multiphoton microscope.

## Declaration of competing interest

The authors declared they do not have anything to disclose regarding conflict of interest with respect to this manuscript.
