# Supplementary material for: Transglutaminase 2 limits the extravasation and the resultant myocardial fibrosis associated with factor XIII-A deficiency
Source: Atherosclerosis. 2020 Feb;294:1–9. doi: 10.1016/j.atherosclerosis.2019.12.013 (PMC7024992; doi:10.1016/j.atherosclerosis.2019.12.013)
Supplement: Multimedia component 1 [file mmc1.docx]

**Transglutaminase 2 limits the extravasation and the resultant myocardial fibrosis associated with Factor XIII-A deficiency**

Kathryn J. Griffin, Laura M. Newell, Kingsley R. Simpson, Cora M.L. Beckers, Mark J. Drinkhill, Kristina F. Standeven, Lih T. Cheah, Siiri E. Iismaa, Peter J. Grant, Christopher L. Jackson, Richard J. Pease

**Supplementary methods**

**Plasma metabolites**

Plasma samples obtained at termination from the mixed strain mice were submitted for automated analysis of lipids by Roche. Plasma samples obtained from the C57BL/6J mice were submitted for automated analysis to the Clinical Biochemistry laboratory of the Leeds Teaching Hospitals NHS Trust. Plasma glucose concentrations from both groups of mice were analysed for glucose using Accu-check Aviva glucometer (Roche Products, UK).

**Insulin and glucose tolerance tests**

Mice of the ages stated were fasted either overnight (for glucose tolerance) or for 4h (insulin tolerance) and then either glucose (1 mg/g body weight) or insulin (0.75 IU/g body weight, Actrapid, Novo Nordisk, UK) was introduced by intraperitoneal injection. Blood glucose measurements were determined from a single tail bleed at baseline and at 30, 60, 90 and 120 minutes post challenge.

**Assays of macrophage function**

Bone marrow was flushed from the long bones of C57BL/6J WT mice and C57BL/6J *Tgm2*/*F13a1* double knockout mice. Macrophages were cultured in RPMI 1640 medium containing 20% foetal bovine serum plus glutamine and supplemented with recombinant murine macrophage colony stimulating factor (MCSF, 50ng/mL, eBioscience) with or without interleukin 4 (10ng/mL). To assess phagocytosis, 5x10^6^ Alexa Fluor 488 conjugated Zymosan A bioparticles (Life Technology) were added to 5x10^4^ adherent macrophages for up to 120 minutes, before washing with phosphate buffered saline, fixing in 4% formaldehyde in phosphate buffered saline (37°C pH 7.4), and mounting in Vectashield with DAPI (Vector Laboratories). To assess cell migration, 0.5x10^6^ macrophages were layered onto gelatin-coated filters (8µm pore, Corning 24 well format). Cells were incubated for up to 16h, fixed, and then non-migrated cells were wiped from the upper face of the membrane with a cotton bud. Filters were excised, mounted in Vectashield with DAPI (Vector Laboratories) and migrated cells on the lower face were counted.

**Vascular permeability protocol**

C57BL/6J mice of each transglutaminase genotype were briefly anaesthetised and then 0.1mL of 1% Evans Blue dye (Sigma) in buffered isotonic saline was injected into the tail vein. After 30 min, the mice were re-anaesthetised, and perfused with 10mL of 4% formaldehyde in phosphate-buffered saline through the left cardiac ventricle. Aortas, and in some cases also hearts, were excised. Aortas and trimmed cardiac ventricles were dried under vacuum and weighed, prior to extraction of Evans Blue into 1mL of formamide for 24h at 60^o^C. The absorbance at 620nm was measured against a standard curve to quantify the amount of Evans Blue extracted and this value was corrected for the tissue dry weight.^1^

**Cardiac pressure-volume loop measurements**

Cardiac function was assessed using pressure volume loops in C57BL/6J WT and *Tgm2*/*F13a1* double knockout mice that had been maintained on a standard chow diet. Mice were anaesthetised with isoflurane (5% induction, 1–1.5% maintenance) and oxygen and placed on a heating pad maintained at 37 ± 1°C. Using an OPMI Pico surgical stereomicroscope (Carl Zeiss), a 1.4 F high-fidelity pressure-conductance catheter (SPR-839; Millar Instruments, Houston, TX, USA) was inserted through the right carotid artery and then into the left ventricle via the aortic valves. This was connected to a pressure-conductance unit (MPVS-300; Millar Instruments) and the data acquired using a Power lab 8/30 (AD instruments) attached to a computer running LabChart 7 Pro software (AD instruments). After 10 minutes of stabilisation, baseline pressure-volume loops were recorded and analysed using PVAN 3.6 software (Millar Instruments) to determine cardiac haemodynamics. Parallel conductance of surrounding tissue was calculated by injecting a bolus (3µL) of hypertonic saline into the left jugular vein. Before cardiac catheterisation, the Millar device was calibrated for pressure using a mercury manometer. At the end of the experiment, fresh heparinized blood was collected from the animal and used to fill a cuvette to calibrate for volume. At the termination of the experiment, hearts were excised and examined for the presence of fibrosis.

**Thromboelastometry**

Blood was drawn from the inferior vena cava, adjusted to 10mM sodium citrate and stored for <1h at 0^o^C until use. Whole blood (105µl) was pipetted into a mini-cup, recalcified to 14mM CaCl_2,_ then clotting was initiated with 7 µL of INTEM reagent (rabbit brain thromboplastin preparation, Tem Ltd, UK). The reaction was followed at 37^o^C for 2 hours by rotational thromboelastometry using the ROTEM® delta system (Tem Ltd, UK).

**Carotid ligation**

Twelve 8-12 week old female mixed strain *Apoe* knockout mice of each transglutaminase genotype underwent left carotid artery ligation under 2.5% isoflurane anaesthesia.

No bleeding presented either during the surgery or immediately post-operatively. Four weeks after surgery, surviving mice were perfusion fixed before excision of the left carotid artery. Consecutive paraffin cross-sections (3 μm) along the carotid artery were taken every 60μm from the ligature – which was designated “0 μm” – until the appearance of the aortic arch, and were stained with EVG and H&E for morphometric analysis.

**References**

[1] Orr, AW, Stockton, R, Simmers, MB, et al., Matrix-specific p21-activated kinase activation regulates vascular permeability in atherogenesis, J. Cell Biol, 2007;176:719-727.

[2] Kumar, A and Lindner, V, Remodeling with neointima formation in the mouse carotid artery after cessation of blood flow, Arterioscler. Thromb. Vasc. Biol, 1997;17:2238-2244.


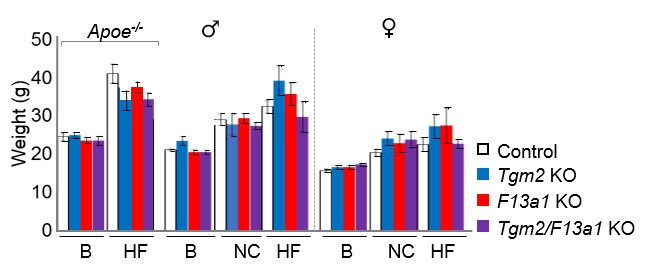


**Supplementary Figure I.** **Growth of *Tgm2* and/or *F13a1* knockout mice**

Body weights were determined at baseline (B, 8 weeks old), and after feeding a normal chow (NC) or high-fat (HF) diet for an additional 12 weeks. Results are shown for male mixed strain *Apoe* knockout (*Apoe*^-/-^) mice (n>20 per group) and for both male and female C57BL/6J *Apoe*-expressing mice (n=6-10). In each case, the control group has the same *Apoe* status as the *Tgm* knockout mice. Weight gain upon fat feeding was less amongst the *Apoe*/*Tgm2* double knockout than *Apoe* single knockout mice and less in *Apoe*/*Tgm2*/*F13a1* triple knockout mice than *Apoe*/*F13a1* double knockout mice, (p<0.01 in each case). However, weight gain was not reduced in *Apoe*-expressing mice following *Tgm2* knockout.


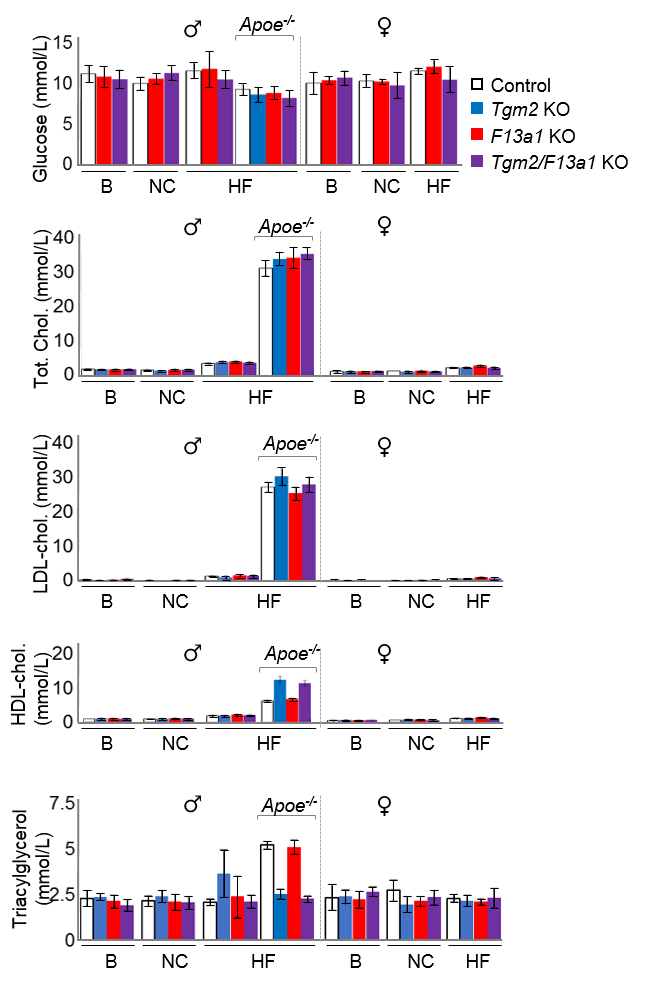


**Supplementary Figure II.** **Plasma** **metabolite levels of *Tgm2* and/or *F13a1* knockout mice.**

Plasma metabolite levels were determined for male mixed strain *Apoe^-/-^* mice (n>20) after 12 weeks of fat-feeding. Plasma metabolite levels were determined in male and female C57BL/6J *Apoe*-expressing *Tgm* knockout mice, (n=6-10 per group) at baseline (B, 8 weeks old) and then after feeding a normal chow (NC) or high-fat (HF) diet for an additional 12 weeks. For both cohorts of mice, the control group has the same *Apoe* status as the *Tgm* knockout (KO) mice. After fat-feeding, *Apoe*/*Tgm2* showed reduced triacylglycerol levels relative to *Apoe* knockout mice (p<0.01) while *Apoe*/*Tgm2*/*F13a1* knockout mice showed reduced triacylglycerol levels relative to *Apoe*/*F13a1* knockout mice (p<0.001). *Apoe*/*Tgm2* knockout mice showed elevated HDL levels relative to *Apoe* knockout mice (p<0.01), similarly *Apoe*/*Tgm2*/*F13a1* knockout mice showed increased high density lipoprotein cholesterol (HDL chol) levels relative to *Apoe*/*F13a1* knockout mice (p<0.001). Glucose, total (tot) cholesterol and low density lipoprotein (LDL) cholesterol did differ not significantly between transglutaminase genotypes in *Apoe* knockout mice. Metabolite levels did not differ between transglutaminase genotypes in the *Apoe* expressing mice.


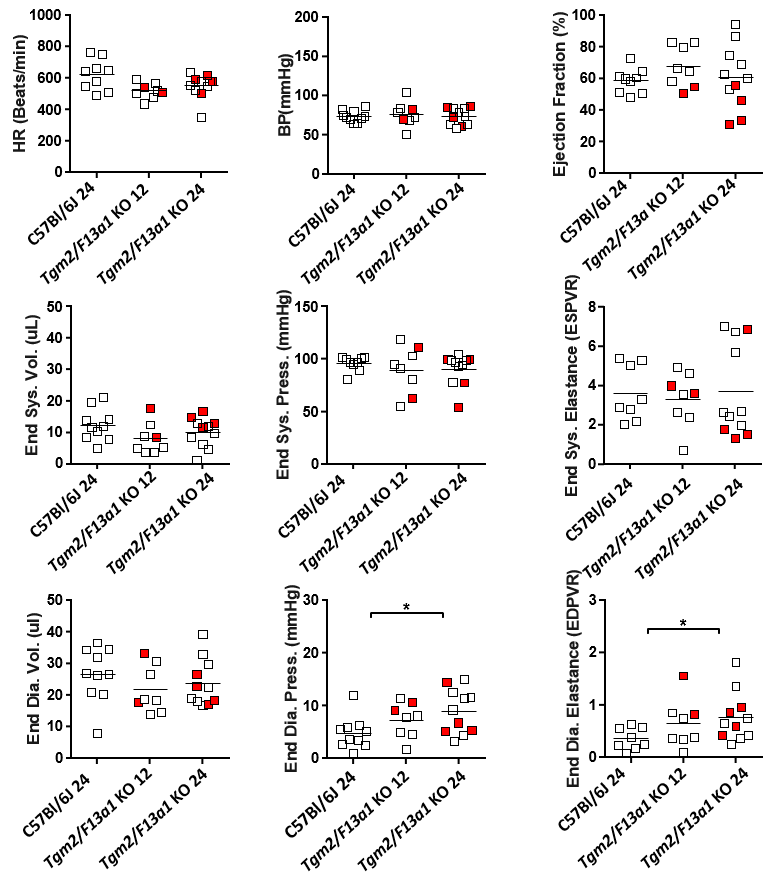


**Supplementary Figure III**. **Cardiac function in *Apoe* expressing C57Bl/6J mice and C57Bl/6J *Tgm2*/*F13a1* double knockout mice**

Pressure-volume loop parameters were measured in anaesthetised C57BL/6J wild-type (WT) at 24 weeks and *Tgm2*/*F13a1* knockout (KO) mice at age 12 and 24 weeks. Subsequently, mouse hearts were excised to determine the degree of ventricular fibrosis. Wild-type mice remained free of fibrosis, while those *Tgm2*/*F13a1* knockout mice that developed the highest amount of fibrosis per age group are indicated red in the cardiac function plots. Heart rate and mean blood pressure were similar between the groups. Mean ejection fraction did not vary between the groups although some 24 week fibrotic mice had a low ejection fraction suggesting incipient heart failure. End elastance in systole and diastole was calculated as (end pressure / end volume). End diastolic elastance was at the expected value in wild-type mice, but was significantly elevated in *Tgm2*/*F13a1* knockout mice consistent with the onset of fibrosis (** p< 0.05*).

**
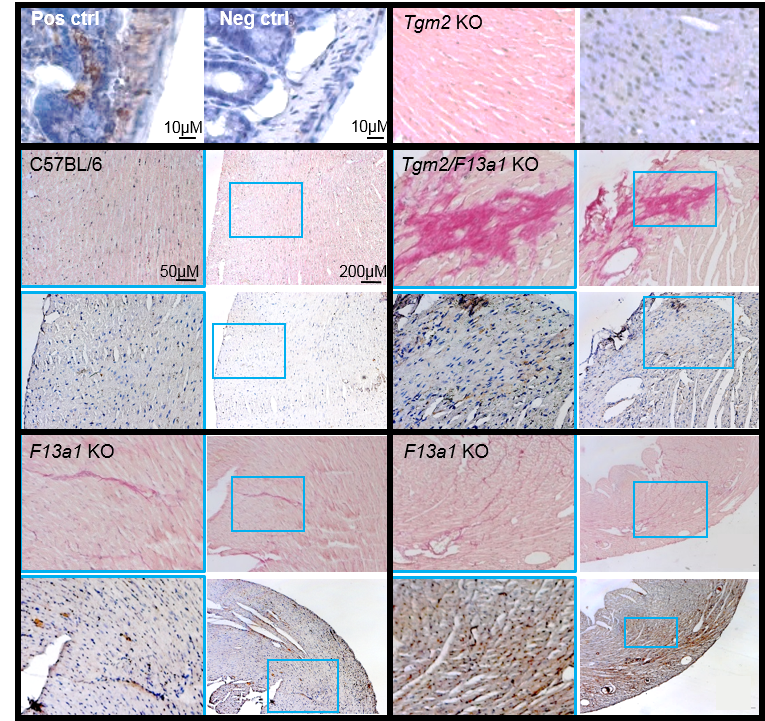
**

**Supplementary Figure IV. Variable TGF-β staining in fibrotic regions of the heart from *Apoe* expressing C57BL/6J mice deficient in transglutaminases TG2 and/or FXIII-A.**

Panel (A) shows the presence of TGF-β antigen (detected as DAB reaction product, brown) in the recommended positive control (Pos ctrl) tissue, mouse colon. DAB reaction product was not detected in the absence of primary antibody (Neg ctrl). Panels (B), (C), (D) and (E) show the distributions of fibrosis determined by Picosirius red staining (upper row) and of TGF-β antigen (lower row) in mice of the genotypes indicated. Scale bars shown for the WT C57BL/6J mouse apply to all myocardial images. DAB reaction product was not detected in myocardial sections in the absence of the primary antibody (not shown). Immunohistochemistry reveals minimal staining for TGF-β in non-fibrotic hearts from WT mice (B). Staining was variable in *F13a1* knockout hearts in some cases indicating small deposits of antigen proximal to areas of fibrosis (C) but in another case showing regions of intense staining plus diffuse staining over a wider area (D), suggesting an ongoing pro-fibrotic insult. Intense staining was also apparent in regions proximal to fibrotic regions in *Tgm2*/*F13a1* double knockout mice (E). Panel (F) shows details of (left) Picrosirius red staining and (right) TGF-β antigen from a *Tgm2* knockout mouse which, as expected, was not fibrotic and showed minimal staining for TGF-β.


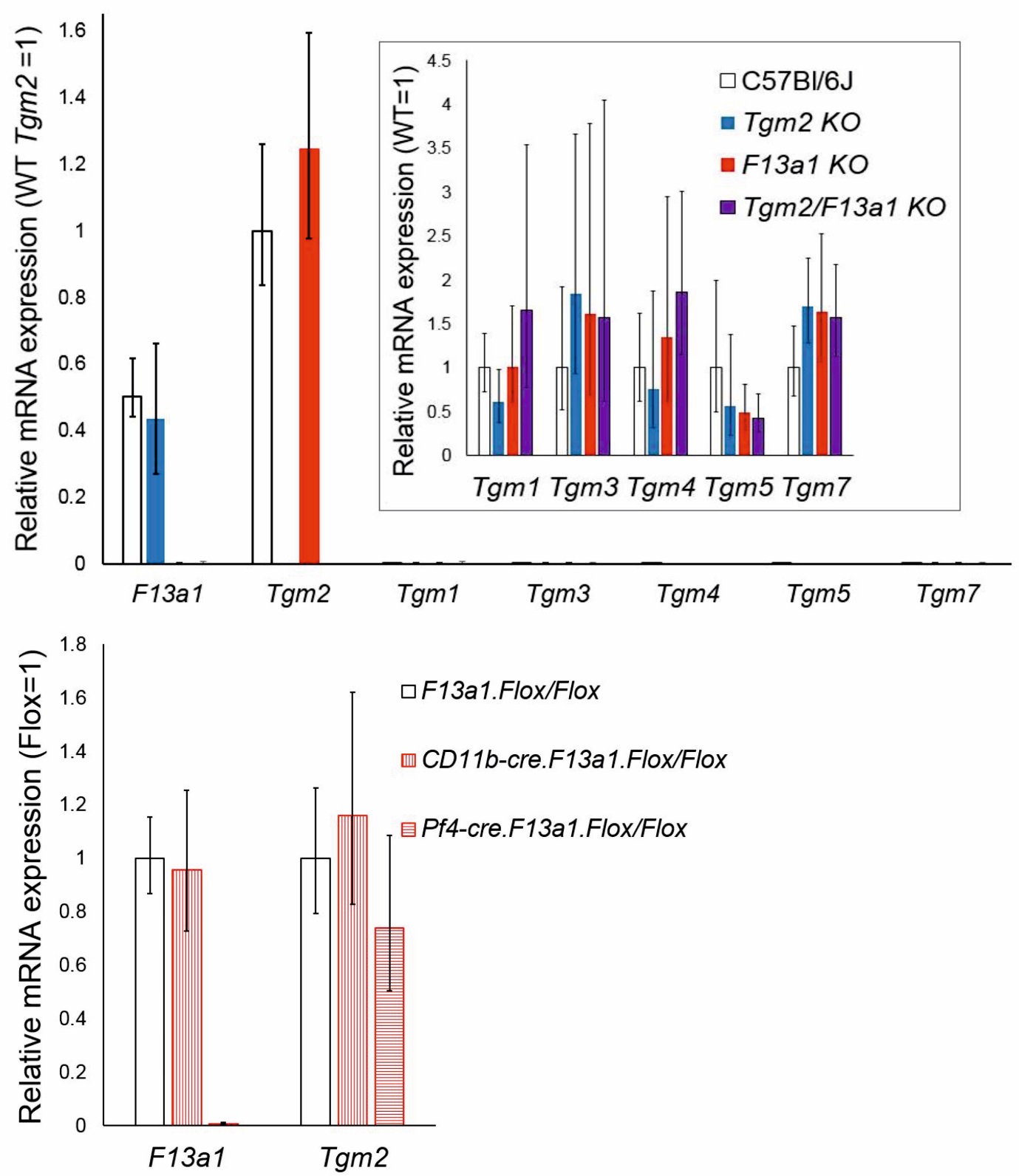


**Supplementary Figure V. Transglutaminase mRNA levels in hearts from *Apoe* expressing C57BL/6J mice deficient in TG2 and/or FXIII-A.**

Transcripts encoding the various transglutaminases (Tgm) were determined by quantitative PCR and adjusted to Rpl32 mRNA. Panel (A) shows levels of transcripts encoding each transglutaminase in the *Tgm* knockout mice (n=10) expressed as a proportion of *Tgm2* mRNA in wild-type C57BL/6J mice (n=20). Transcripts encoding FXIII-A and TG2 were abundant except in the relevant knockout mice (Inset). Transcripts encoding Tgms1, 3, 4, 5 and 7 (but not 6) were detectable at low levels within heart. The levels of each transcript in *Tgm* knockout mice is shown as a proportion of that *Tgm* mRNA in WT mice. Changes in level of these other *Tgm* mRNAs were small and not expected to alter total transglutaminase activity within the heart. Panel (B) shows transcript levels encoding FXIII-A and TG2 in hearts from *Pf4*-cre.F13a1^flox/flox^ mice and *Cd11b*-cre.F13a1^flox/flox^ mice relative to levels in F13a1^flox/flox^ mice, (n=10).


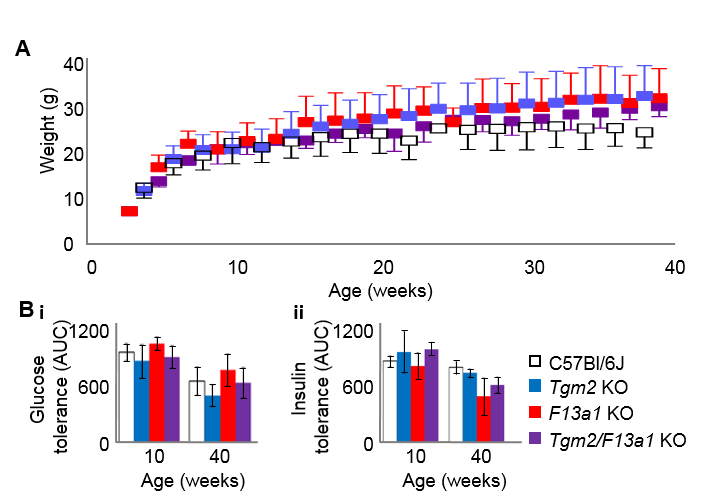


**Supplementary Figure VI.** **Growth and glucose metabolism in *Apoe* expressing** **C57BL/6J mice deficient in TG2 and/or FXIII-A**

Panel (A) shows body weight up to 40 weeks in C57BL/6J mice (WT, wild-type) and C57BL/6J Tgm knockout mice. There was no significant difference in weight gain between the genotypes. Panel (B) shows (i) glucose and (ii) insulin tolerance in mice of 10 weeks of age (n=8-10 per genotype) and 40 weeks of age (n=8-11, except for *F13a1* KO where n=4), based upon venous glucose concentrations measured. In each case, the area under the curve (AUC) was determined from a plot of glucose concentration against time and has dimensions of concentration.time (mmol.L^-1^.min).


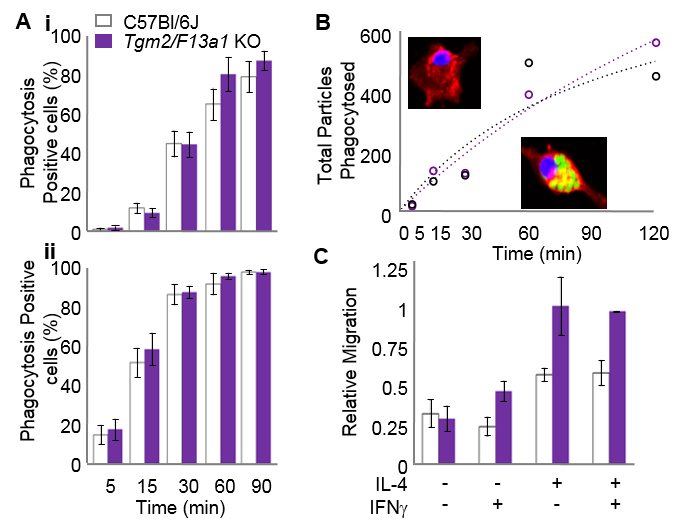


**Supplementary Figure VII.** **Functionality of macrophages from *Apoe* expressing C57BL6/J** **wild-type and *Tgm2*/*F13a1* double knockout mice.**

Panel (A) shows the percentage of bone marrow-derived macrophages that scored positive for uptake of labelled Zymosan A bioparticles, as a function of time following pre-incubation with (Ai) or without (Aii) IL-4 (n=4 independent experiments). Panel (B) shows the number of particles phagocytosed in a field of approximately 50 cells, averaged from 2 representative experiments in panel (A). The inset show images of an ‘empty’ macrophage (cytoskeleton (red), nucleus (blue)) at the start of incubation, and a replete macrophage following engulfment of bioparticles (green). Neither the percentage of cells positive for one or more particles (A) nor the total number of engulfed bioparticles (B) varied between wild-type (WT) and *Tgm2*/*F13a1* knockout (KO) mice. Panel (C) shows the relative numbers of bone marrow-derived macrophages that had migrated through a porous filter (8µm) during 16h of incubation in the presence of interleukin-4 (IL-4) and/or interferon γ (IFN-γ). In the presence of IL4 and/or IFN-γ *Tgm2*/*F13a1* knockout macrophages appeared to migrate faster than wild-type macrophages (n=4 independent experiments). The relative migration of macrophages from mice of differing genotypes was similar after incubation for 2h or 4h (not shown).


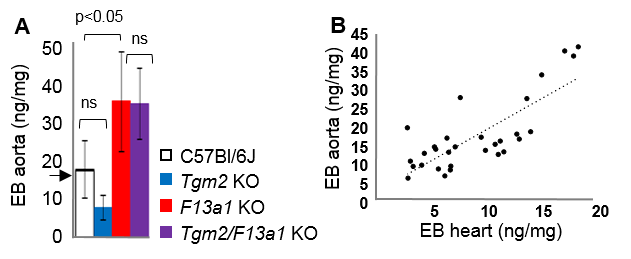


**Supplementary Figure VIII.** **Increased vascular permeability in *Apoe* expressing *F13a1* knockout mice.**

Panel (A) shows the amount of Evans Blue (EB) dye extracted from perfused and excised mouse aortas 30 minutes after intravenous injection of the dye, and normalised for the dry weight of the tissue. Mean uptake into the aortas of C57BL/6J wild-type (WT) mice was in close agreement with published values^1^ (arrowhead), and was increased in both *F13a1* knockout (KO) and *Tgm2*/*F13a1* double knockout mice (n=10-12 per group). Panel (B) shows that the amount of Evans Blue dye that accumulated in aorta strongly correlated with that in heart (r=0.830) in those animals from which both tissues were extracted (n=30).


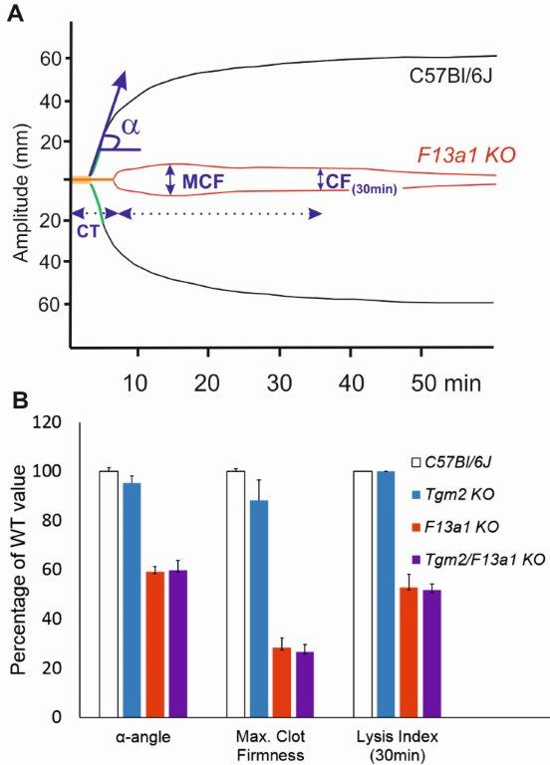
**Supplementary Figure IX. Clotting parameters in *Apoe* expressing mice measured by thromboelastometry.**

Panel (A): The time-course of clotting of recalcified and thromboplastin-treated whole blood from C57BL/6J WT and C57BL/6J *F13a1* knockout mice was followed using the ROTEM® analyser. The α-angle represents Δ_amplitude_/time from the clotting time (CT, shown above for the *F13a1* KO mouse) to an amplitude of 20mm. The α-angle is reduced in *F13a1* KO mouse, presumably because lysis occurs alongside clotting. The maximum clot firmness (MCF) is given by maximum amplitude difference over the clotting profile and reaches a plateau in the WT mouse but decreases in the *F13a1* KO mouse due to lysis. The lysis index (LI) at 30 min is given by the clot firmness (CF) measured 30 min from the onset of clotting divided by maximum clot firmness (CF_30_/MCF). Panel (B): The average value (n=6) of each clotting parameter in mice of the genotypes shown is expressed as a percentage of that parameter in C57BL/6J wild-type mice. *F13a1* knockout mice showed a decrease in the α-angle, the maximum (max) clot firmness and the lysis index, but no additional decrease in these parameters was observed in *Tgm2*/*F13a1* double knockout mice.


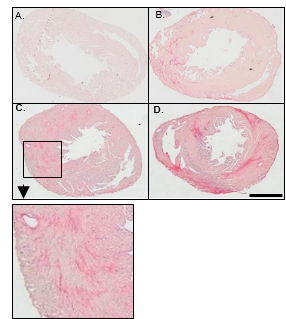


**Supplementary Figure X. FXIII-A in inflammatory, but not resident cardiac, macrophages may confer protection against fibrosis**

Picrosirius red staining of hearts from (A) *a Pf4-cre.F13a1 ^flox/flox^* mouse, (B) a *F13a1* knockout mouse, (C) a *Cd11b*-cre.*F13a1*^flox/flox^ mouse and (D) a *Tgm2*/*F13a1* double knockout mouse. While fibrosis was not apparent in *Pf4*-cre.FXIII-A^flox/flox^ mice, fibrosis was evident in *Cd11b*-cre.FXIII-A^flox/flox^ mice (iii and detail), suggesting that FXIII-A within inflammatory macrophages may be important to limit fibrosis. Scale bar represents 1000µm.


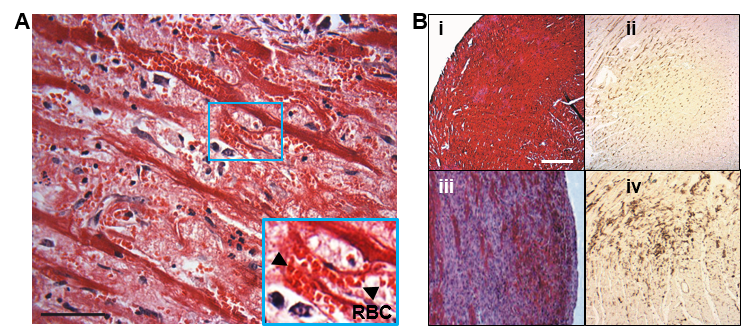


**Supplementary Figure XI. Occasional presence of red blood cells and inflammatory macrophages within the myocardium of *Apoe*/*Tgm2*/*F13a1* triple knockout mice.**

Panel (A) shows a section from a perfusion-fixed mixed strain *Apoe*/*Tgm2*/*F13a1* knockout mouse. Clusters of red blood cells (RBC) are apparent (indicated with arrowheads in the area detailed) throughout this region of the myocardium. Panel (B) shows (i) a non-fibrotic region of ventricle from an *Apoe*/*Tgm2*/*F13a1* triple knockout mouse stained with Masson’s trichrome (M). (ii) Light regular staining of cells was apparent in the non-fibrotic area after incubating with GSL lectin. In a fibrotic region from the same mouse (iii), intense irregular staining was apparent after incubation with GSL lectin (iv), indicating recent macrophage infiltration into this area.


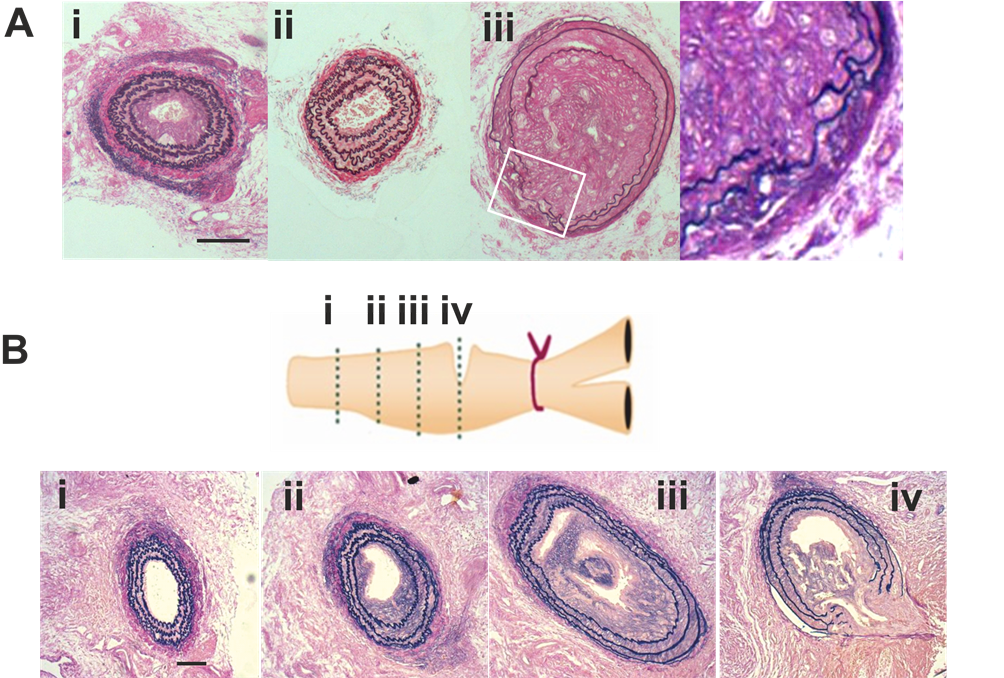


**Supplementary Figure XII. Carotid ligation leads to elastic breakage in mixed strain *Apoe*/*Tgm2* double knockout mice and to vessel rupture in *Apoe*/*Tgm2*/*F13a1* triple knockout mice.**

Carotid arteries were ligated in *Apoe* knockout, *Apoe*/*F13a1* double knockout, *Apoe*/*Tgm2* double knockout and *Apoe*/*Tgm2*/*F13a1* triple knockout mice with the expectation of inducing and quantifying atherosclerosis.^2^ Panel (A): The expected outcome of inward remodelling was observed in *Apoe* (i) and *Apoe*/*F13a1* knockout mice (ii). In *Apoe*/*Tgm2* double knockout mice, vessel dilatation occurred (panel iii) associated with elastic breaks (detail). The frequency of elastic breaks per section increased in the *Apoe*/*Tgm2* double knockout mice (*Apoe* knockout 5.4±1.6, *Apoe*/*F13a1* double knockout 4.3±1.4, *Apoe*/*Tgm2* double knockout 16.2±2.12, n=12 per genotype, p<0.01). Panel (B): Carotid ligation led to vessel rupture in 6/12 *Apoe*/*Tgm2*/*F13a1* triple knockout mice, but not in mice of other genotypes, (p<0.01). Panels (i), (ii), (iii), and (iv) show sections at 60μm intervals as illustrated on the schematic diagram. Scale bar represents 100µm.

**
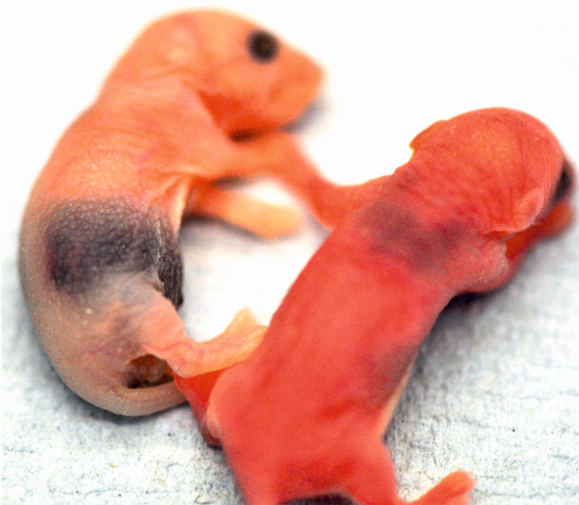
**

**Supplementary Figure XIII. Abdominal haemorrhage in *Apoe* expressing *F13a1* knockout mouse pups from rare litters.**

The image shows *F13a1* knockout and *F13a1*^+/-^ heterozygous pups that were born following mating an apparently normal C57BL/6J *F13a1^+/-^* female with an apparently normal C57BL/6J *F13a1*^-/-^ male. In the litter of 8 pups, 5 showed haemorrhage of the abdominal wall and pallor of the thorax and head, while 3 pups were healthy. Genotyping confirmed that the affected pups were homozygous *F13a1* knockout mice, while the viable pups were heterozygous.

| **Gene** | **Target Allele** | **Sequence (5’-3’)** |
| --- | --- | --- |
| **Mixed strain C57BL/6J/129 mice** | | |
| *Apoe* | KNOCK-OUT | CGCCGCTCCCGATTCGCAGCGCATCGC |
| *Apoe* | WILD TYPE | CTCTGTGGGCCGTGCTGTTGGTCACATTGCTGACA |
| *Apoe* | COMMON | CTCGAGCTGATCTGTCACCTCCGGCTCTCCC |
| **Mixed strain and C57BL/6J *Tgm2* knockout mice** | | |
| *Tgm2* | KNOCK-OUT | GGAGCACACAGGCCTTATGAGCTGAAG |
| *Tgm2* | WILD TYPE | GCACAGATAGGGATACAAGAAGCATTGAAG |
| *Tgm2* | COMMON | GTCCTCGGATGACAAGGTGACAGAGCA |
| **Mixed strain *F13a1* knockout mice** | | |
| *F13a1* | KNOCK-OUT | CACTGCATTCTAGTTGTGGTTTGTCCAAACTC |
| *F13a1* | WILD TYPE | CAGGCTCCCCTGAGACTTACGGATGAAG |
| *F13a1* | COMMON | CCAAGGATGATGAAGGTGTTCTTGTTGG |
| **C57BL/6J *F13a1* knockout mice** | | |
| *F13a1* | KNOCK-OUT | GGGGACTTGCTCCCATGTAAA |
| *F13a1* | WT & FLOX | CAAGACCAGACTGTGCAAAGGG |
| *F13a1* | COMMON | TCTGGGCCAAACCAAGTACCTGG |
| **C57BL/6J *–cre* mice** | | |
| *Pf4* | WT & TRANSGENE | TGGGCAGGCAGTGAAGATAA  CATGTCAAGAGGGTGCCACTGGA |
| *Cd11b* | TRANSGENE | CGACCAGGTTCGTTCACTCA  CAGCGTTTTCGTTCTGCCAA |

**Supplementary Table 1:** PCR primer sequences for genotyping

| **Gene** | **Forward Primer** | **Reverse Primer** |
| --- | --- | --- |
| *Elastin* | AAAGCCTGGGAAAGTTCCTG | TACACCTGGAAGACCAACAC |
| *Col1a1* | ATGGATTCCCGTTCGAGTACG | TCAGCTGGATAGCGACATCG |
| *Col3a1* | CACCCTTCTTCATCCCACTCTTA | ACCAAGGTGGCTGCATCC |
| *Vimentin* | CGGAAAGTGGAATCCTTGCA | CACATCGATCTGGACATGCTGT |
| *A2Sma* | ACTGGGACGACATGGAAAAG | GTTCAGTGGTGCCTCTGTCA |
| *MMP2* | CTGATAACCTGGATGCCGTCGT | TGCTTCCAAACTTCACGCTCTT |
| *MMP9* | GTCTCGGGAAGGCTCTGCTGTT | CTCTGGGGATCCACCTTCTGAG |
| *F13a1* | TGCTGGTGTCTTTAACACATTTTTAA | TGGGCCGAGAAGTAATTGGT |
| *Tgm1* | TTCGCTACCCGTACCGTCA | CTTCATCCAGCAGTCGTT |
| *Tgm2* | ATTGGCAGTGTGGACATTC | TCGTGGGCGGAGTTGTA |
| *Tgm3* | AAGAAGCTGACCATGAGTGCTTT | TGCGCCCTTCGATTCATAG |
| *Tgm4* | CCCATCTATTTGACCATAACTTTGAA | GTGAGAAACACCCTTGATT |
| *Tgm5* | AGGCAGGATTCTGGAGAATATG | TAGAGACCATTGCTGGTTTCCTG |
| *Tgm6* | TCCGAGTCAATGTGAGCG | GTCTTCTGTCAGGTCTCCTTTGTA |
| *Tgm7* | ATGTGCACGGTAATGAGATGCT | TGTGTGCAGAATGGAAATTGG |
| *CD163* | ATGGGTGGACACAGAATGGTT | CAGGAGCGTTAGTGACAGCAG |
| *tPA (plat)* | CAACAGCGGCCTGGTACAA | CCCCATTGAAGCATCTTGGTT |
| *uPA*  *(plau)* | GAAACCCTACAATGCCCACAGA | GACAAACTGCCTTAGGCCAATC |
| *PAI-1*  *(serpine2)* | ACGGTGATGCGATATAATGTAAACG | CATTCCTGAGAAACACAGCATTG |
| *Fbn1* | CCTGTGCTATGATGGGTTCA | AGGTCCCACTAAGGCAGATG |
| *Fbn2* | CCACTCCTATTGCTGCCCAG | TTGGGGCGGGAACAGAATC |
| *MT-MMP (MMP14)* | CCCAAGGCAGCAACTTCA | CAATGGCAGCTGAGAGTGAC |
| *Hmox1* | AACAAGCAGAACCCAGTCTATG | TGAGCAGGAAGGCGGTCTTA |
| *MMP12* | GCTAGAAGCAACTGGGCAAC | ACCGCTTCATCCATCTTGAC |
| *TIMP1* | GTGGGAAATGCCGCAGAT | GGGCATATCCACAGAGGCTTT |
| *TIMP2* | CCAGAAGAAGAGCCTGAACCA | GTCCATCCAGAGGCACTCATC |
| *Tgfb1* | CACCGGAGAGCCCTGGATA | TGTACAGCTGCCGCACACA |
| *B-Actin* | CGTGAAAAGATGACCCAGATCA | TCGTACGACCAGAGGCATACAG |
| *RPL32* | AAAATTAAGCGAAACTGGCGG | TGTTGCTCCCATAACCGATG |
| *Cd11*  *Itgam* | GACCGTCTGCGCGAAGGAGATA | CGCCTGCGTGTGTTGTTCTTTG |
| *Man Rec*  *MRC1* | CTCTGTTCAGCTATTGGACGC | CGGAATTTCTGGGATTCAGCTTC |
| *VWF* | GCTTGAACTGTTTGACGGAGAGG | TGACCCAGCAGCAGGATGA |

**Supplementary Table 2:** Primer sequences for quantitative PCR

| **Study 1: *Apoe* knockout mice in each group: *F13a1* knockout achieved with the Lauer *et al* mouse** | | | | | |
| --- | --- | --- | --- | --- | --- |
| **Genotype**  (All mice on a C57BL/6J x SVJ129 background) | **Atherosclerosis after 12 weeks HF diet**  **(Table 1)** | | | **Cardiac fibrosis after HF diet** **(Figure 1)** | **Metabolic/physiological parameters** |
| ***Apoe^-/-^* (♂) = Control** | BCA positive  AS positive | | | Negative | - Weight gain on HF diet different between groups (SF I) - Plasma triacylglycerol and HDL-cholesterol levels after HF diet different between groups (SF II) - Haemosiderin (Figure 1c) and red blood cells (SF XI) detected in myocardium of fibrotic mice - High mortality occurred in *Apoe^-/-^/F13a1^-/-^* and *Apoe^-/-^Tgm2^-/-^/F13a1^-/-^* mice maintained on a HF diet - *F13a1 ^-/-^* mice complete pregnancy - Elastic lamellae of *Apoe^-/-^Tgm2^-/-^ and Apoe^-/-^Tgm2^-/-^/F13a1^-/-^* mice break upon arterial ligation (SF XII) |
| ***Apoe^-/-^/Tgm2^-/-^* (♂)** | BCA plaque area ≈ Control plaque area  AS plaque area ≈ Control plaque area | | | Negative |  |
| ***Apoe^-/-^/F13a1^-/-^* (♂)** | BCA plaque area ≈ Control plaque area  AS plaque area ≈ Control plaque area | | | Minimal (0.4%) |  |
| ***Apoe^-/-^Tgm2^-/-^/F13a1^-/-^* (♂)** | BCA plaque area < Control plaque area  BCA density of buried caps ≈ Control density  AS plaque area ≈ Control plaque area | | | Extensive (12%) |  |
| **Study 2: *Apoe* expressing mice in each group: *F13a1* knockout achieved with the Beckers *et al* *F13a1* KO or *F13a1flox/flox* mouse.** | | | | | |
| **Genotype**  (All mice on a C57BL/6J background) | **Athero-sclerosis** | **Cardiac fibrosis on HF or NC diet** **(Figure 2)** | | | **Metabolic/physiological parameters** |
|  |  | **Baseline at 8 weeks old** | **After 12 weeks of feeding** | | - Weight gain and metabolites on NC diet similar between genotypes (SF I & II) - Weight gain and metabolites on HF diet similar between genotypes (SF I & II) - Transcript levels in young mice similar between genotypes (Figure 4) - Insulin & glucose tolerance similar between genotypes (SF VI) - Phagocytosis similar between WT and *Tgm2^-/-^/F13a1^-/-^* mice (SF VII) - Endothelial permeability increased in *F13a1^-/-^* and *Tgm2^-/-^/F13a1^-/-^* mice (SF VIII) - *F13a1^-/-^* mice do not complete pregnancy. - Haemosiderin correlates with fibrosis within the myocardium (Figure 2) - *Tgm2^-/-^/F13a1^-/-^* mice show altered ventricular function (Figure 2, SF III) - *F13a1 ^-/-^ and Tgm2^-/-^/F13a1^-/-^* mice survive HF diet. - Red blood cells detected in the myocardium of a fibrotic mouse (Figure 3). |
| **WT (♂ & ♀) and**  ***Tgm2^-/-^* (♂ & ♀)** | nd | HF Negative & NC Negative | | |  |
| ***F13a1^-/-^* (♂ & ♀)** | nd | Minimal | ♂ (4%) > ♀ | |  |
| ***Tgm2^-/-^/F13a1^-/-^* (♂ & ♀)** | nd | Apparent  ♂ (4%) > ♀ | Extensive  ♂ (15%) > ♀  Fibrosis is interstitial (Figure 3) | |  |
| ***Pf4-cre.F13a1flox/flox* (♂ & ♀)** | nd | NC negative | NC negative | | - Absence of FXIII-A in macrophages. - Depletion of plasma FXIII-A to 20% of WT |

SF = Supplementary figure
